# Municipality-Level Predictors of COVID-19 Mortality in Mexico: A Cautionary Tale

**DOI:** 10.1017/dmp.2020.485

**Published:** 2020-12-22

**Authors:** Alejandra Contreras-Manzano, Carlos M. Guerrero-López, Mercedes Aguerrebere, Ana Cristina Sedas, Héctor Lamadrid-Figueroa

**Affiliations:** 1 Center for Nutrition and Health Research, National Institute of Public Health, Cuernavaca, Morelos, Mexico; 2 Mexican Social Security Institute, Juárez, Cuauhtémoc, Mexico; 3 National Autonomous University of Mexico, Mexico City, Mexico; 4 Department of Global Health and Social Medicine, Harvard Medical School, Boston, Massachusetts USA; 5 Center for Population Health Research, National Institute of Public Health, Cuernavaca, Morelos, Mexico

**Keywords:** COVID-19, risk factors, social determinants, health determinants, municipality-level

## Abstract

**Objective:**

Local characteristics of populations have been associated with coronavirus disease 2019 (COVID-19) outcomes. We analyze the municipality-level factors associated with a high COVID-19 mortality rate (MR) of in Mexico.

**Methods:**

We retrieved information from cumulative confirmed symptomatic cases and deaths from COVID-19 as of June 20, 2020, and data from most recent census and surveys of Mexico. A negative binomial regression model was adjusted, the dependent variable was the number of COVID-19 deaths, and the independent variables were the quintiles of the distribution of sociodemographic and health characteristics among the 2457 municipalities of Mexico.

**Results:**

Factors associated with high MRs from COVID-19, relative to quintile 1, were diabetes and obesity prevalence, diabetes mortality rate, indigenous population, economically active population, density of economic units that operate essential activities, and population density. Among factors inversely associated with lower MRs from COVID-19 were high hypertension prevalence and houses without sewage drainage. We identified 1351 municipalities without confirmed COVID-19 deaths, of which, 202 had high and 82 very high expected COVID-19 mortality (mean = 8 and 13.8 deaths per 100,000, respectively).

**Conclusion:**

This study identified municipalities of Mexico that could lead to a high mortality scenario later in the epidemic and warns against premature easing of mobility restrictions and to reinforce strategies of prevention and control of outbreaks in communities vulnerable to COVID-19.

The first case of the new coronavirus disease 2019 (COVID-19) in Mexico was confirmed on February 28, 2020.^1^ Since then, the Government of Mexico has launched a series of preventive measures that adhere to the World Health Organization (WHO) severe acute respiratory syndrome coronavirus 2 (SARS-CoV-2) strategic preparedness and response plan aimed at limiting the spread of the virus.^2^ The National Campaign for Healthy Distance, implemented from March 23 to May 30, 2020, included social distancing, hand washing, general confinement, self-isolation for those with COVID-19 associated symptoms for 14 d, and limited economic mobility.^3^ As of June 20, the spread of the virus continued to rise, resulting in 175,148 accumulated positive cases and 20,773 deaths.^4^

Globally, individual factors associated with COVID-19 mortality have been found to be: male sex, age over 65 y, ethnicity, hypertension, diabetes, cardiovascular disease, and respiratory disease, among others.^5,6^ According to the 2018 National Health and Nutrition Survey (ENSANUT-2018) of Mexico, 36.1% of adults over the age of 19 were obese, 39.1% overweight, 13.7% diabetic, and 25% hypertensive.^7^ Considering that most states in Mexico hold a heterogeneous distribution in the prevalence of such comorbidities, regions with an increased burden of these diseases and sociodemographic-related factors are at higher risk of encountering more severe manifestations of COVID-19, which might require hospitalization or critical care, as well as higher COVID-19 mortality rates (MRs).

It is deemed that, to curb the pandemic, national decisions ought to be in coordination with those at a local level. In the United States, sociodemographic and health factors related to COVID-19 vary importantly across counties.^8-10^ Mexico has 2457 municipalities distributed in 32 states, which are the basis of the territorial organization and the political and administrative division.^11^ By June 1, 2020, the federal government gave the states of the country the responsibility for deciding the reopening of social, educational, and economic activities based on a “traffic light” system established by the Mexican Ministry of Health (MoH).^12^ However, the COVID-19 pandemic is expected to linger within communities for several months or even years to come. Targeted public policy interventions in regions that are highly vulnerable to COVID-19 are crucial to safeguard, protect, and strengthen communities facing the pandemic. Therefore, the objective of this study was to analyze the municipality-level factors associated with a higher MR from COVID-19 in Mexico, and to pinpoint locations expected to suffer from a high COVID-19 mortality.

## Methods

Coronavirus pandemic surveillance in Mexico has been carried out using the Sentinel model proposed by the Pan American Health Organization (PAHO),^13^ implemented in the country in 2019 for the H1N1 pandemic. This model works with 475 nationally representative health facilities that monitor coronavirus cases through testing for SARS-CoV-2, conducted in 10% of suspected cases and 100% of those suspected with SARS and signs of breathing difficulty, or in deaths of those hospitalized suspected to be COVID-19 cases. The operational definition of a suspected case in Mexico is of an individual who in the last 7 d presented at least 2 of the following signs and symptoms: cough, fever, or headache, accompanied by at least 1 of the following signs or symptoms: dyspnea, arthralgia, myalgia, odynophagia/pharyngeal burning, rhinorrhea, conjunctivitis, and/or chest pain.^14^ A confirmed case of COVID-19 is defined as a person with a diagnosis given by the National Network of Public Health Laboratories recognized by the Institute of Epidemiological Diagnosis and Reference (InDRE) who met the criteria of a suspected case.^15^

We used the daily updated open data source from the General Directorate of Epidemiology of the Mexican MoH, which includes demographic and health information of confirmed, negative, and suspected cases of COVID-19.^4^ We extracted the data as of June 20, 2020, of the number of confirmed symptomatic cases and deaths.

Municipality level was defined according to the 2017 geographical division of the country, which included 2457 municipalities in 31 states and 1 federal district.^16^ At the municipality level, we obtained data on variables that we hypothesized could be linked to differential testing practices or exposure to SARS-CoV-2 of the most recent and complete information from census and records from the National Institute of Statistics and Geography (*INEGI* per the Spanish acronym). For the analysis, we selected the following independent variables and available year of collection^17-19^: municipalities’ population size in 2018, proportion of illiteracy in population aged 15 y or more in 2015, proportion of indigenous population of 5 y or more in 2010, proportion of the population over 12 y of age who are economically active in 2015, population with private health insurance in 2015, population affiliated to the Mexican Social Security Institute (IMSS, per the Spanish acronym) in 2015, population without health-care insurance in 2019, proportion of households with indoor availability of water service in 2015, proportion of households without sewage drainage in 2015, population density in 2015, and rate of economic units that operate essential activities during the COVID-19 outbreak in Mexico from the National Directory of Economic Units/INEGI.^20^

Extreme poverty at the municipal level was obtained from the National Council for the Evaluation of Social Development Policy (CONEVAL for its acronym in Spanish).^21^ From the Department of Epidemiological Surveillance of the MoH (DGIS for its acronym in Spanish), we retrieved records of deaths due to diabetes, hypertension, and cardiovascular disease in adults over 20 y of age in 2018.^22^

From the catalog of the Unique Key of Health Establishments (CLUES per the Spanish acronym), we retrieved the number of medical units per municipality level for 2020, and we calculated the rate per 100,000 inhabitants in the municipality for each type of medical unit.^23^ Rates, ratios, and proportions of the variables at the municipality level were calculated using the annual population projections from the National Population Council (CONAPO).^24^ We obtained the state-level estimations for the prevalence of obesity, previously diagnosed diabetes, and previously diagnosed hypertension among adults aged 20 y and older from the latest National Survey of Nutrition and Health (ENSANUT-2018).^25^

All the previously mentioned independent variables of the municipalities were recoded as quintiles of the distribution to provide a better observation of the data. Nevertheless, it is important to mention that, for the original variables, significant associations remain.

### Statistical Analysis

To identify the municipality-level factors associated with the MR from COVID-19, we adjusted a negative binomial regression model in which the dependent variable was the sum of confirmed COVID-19 deaths in symptomatic cases (*n* = 20,773) by municipality of residence and the independent variables were the quintiles of the distribution of sociodemographic and health outcomes within the municipality. Expected MRs (EMRs) for each municipality, incidence rate ratios (IRR), and 95% confidence intervals (CIs) were estimated from the model-predicted case counts divided by the population of the municipality.

To better understand the association between MRs from COVID-19 and municipality-level factors, and in addition to the main objective of the study, we also fitted negative binomial regression models substituting COVID deaths with the cumulative incidence rate of confirmed symptomatic cases (*n* = 142,643) ([cumulative COVID-19 cases/municipal population]*100,000 population), and also we substituted COVID deaths in the model with the case-fatality rate in confirmed deaths from COVID-19 (*n* = 20,773 deaths) ([deaths/confirmed cases]*100) as of June 20, 2020. In a supplementary table, results are displayed stratified by months since the beginning of the pandemic in Mexico.

Finally, to contrast our findings on mortality risk factors with those at individual level in Mexico, we retrieved the following variables from the MoH coronavirus database; age, sex, state, municipality of residence, indigenous languages speaker, diabetes, obesity, hypertension, chronic obstructive pulmonary disease (COPD), cardiovascular disease, chronic kidney disease (CKD), immunosuppression, asthma, dates of symptom onset, hospital admission, and death. We excluded 429 cases as they had incomplete information on the covariates above-mentioned. We fitted a Poisson regression model where the dependent variable was the binary outcome of death due to COVID-19 and the independent variables were the characteristics and binary comorbidities of the individual. The analysis and maps were developed using the statistical program STATA 14 (Stata Statistical Software: Release 14. College Station, TX: StataCorp LP), findings at *P* < 0.05 were considered significant.

## Results

The analyzed sample consisted of 175,148 positive cases of COVID-19 in which 20,773 deaths occurred. At the moment of the analysis, two-thirds of the municipalities of the country reported at least 1 symptomatic case, and almost half of the municipalities reported deaths due to COVID-19 (data not shown).

In Table 1 are displayed the means and 95% CIs of the municipal- and state-level factors included in the study, source of information and year of reference.

**Table 1.** Means of the municipal- and state-level factors included in the study, source of information, and year of reference

Variable	Mean (95% CI)	Source of information	Year	Level
Proportion of population aged 60 y or older	0.20 (0.20, 0.21)	CONAPO/INEGI	2020	Municipal
Proportion of males	0.48 (0.47, 0.48)	CONAPO/INEGI	2020	Municipal
Prevalence of diabetes in adults aged 20 y or older	10.3 (10.2, 10.4)	ENSANUT/INEGI	2018	State
Prevalence of obesity in adults aged 20 y or older	34.5 (28.9, 48.3)	ENSANUT/INEGI	2018	State
Prevalence of hypertension in adults aged 20 y or older	18.3 (18.1, 18.4)	ENSANUT/INEGI	2018	State
Crude MR of diabetes in adults 20 y or older	133.2 (129.5, 136.8)	DGIS/INEGI	2018	Municipal
Crude MR of hypertension in adults 20 y or older	15.7 (14.3, 17.2)	DGIS/INEGI	2018	Municipal
Crude MR of cerebrovascular disease in adults 20 y or older	22.3 (20.6, 24.1)	DGIS/INEGI	2018	Municipal
Proportion of the population in extreme poverty	0.20 (0.19, 0.21)	CONEVAL	2015	Municipal
Proportion of illiterate population aged 5 y or older	0.12 (0.11, 0.12)	INEGI	2015	Municipal
Proportion of indigenous population aged 5 y or older	0.19 (0.18, 0.21)	INEGI	2010	Municipal
Proportion of population economically active aged 12 y or older	0.41 (0.41, 0.42)	INEGI	2015	Municipal
Rate of economic units classified as essential during COVID-19 outbreak	0.03 (0.03, 0.03)	INEGI	2020	Municipal
Population density	292.7 (245.6, 339.7)	INEGI	2015	Municipal
Proportion of houses with water service inside the domicile	0.07 (0.06, 0.07)	INEGI	2015	Municipal
Proportion of houses without sewage drainage service	0.19 (0.18, 0.20)	INEGI	2015	Municipal
Proportion of houses with dirt floor	0.08 (0.08, 0.09)	INEGI	2015	Municipal
Rate of hospitals	2.39 (2.21, 2.57)	CLUES catalog	2020	Municipal
Rate of units of health care services	58.8 (56.6, 61.0)	CLUES catalog	2020	Municipal
Rate of social assistance medical units	3.65 (2.8, 4.49)	CLUES catalog	2020	Municipal
Proportion of population without health-care insurance (was affiliated to the People’s Health Care)	0.64 (0.63, 0.64)	INEGI	2019	Municipal
Proportion of population affiliated to the Mexican Social Security Institute (IMSS)	0.14 (0.14, 0.15)	INEGI	2015	Municipal
Proportion of population with Private Health Insurance	0.01 (0.01, 0.01)	INEGI	2015	Municipal

IMSS: Mexican Social Security Institute.

CONAPO: National Population Council.

INEGI: National Institute of Statistics and Geography.

CLUES: Catalog of the Unique Key of Health Establishments.

ENSANUT: National Health and Nutrition Survey.

CONEVAL: National Council for the Evaluation of Social Development Policy.

DGIS: Department of Epidemiological Surveillance of the Ministry of Health.

Municipality-level factors associated with high mortality from COVID-19 were the prevalence of diabetes (quintile 4; IRR = 3.43; 95% CI, 1.75-2.98), and obesity (quintile 5; IRR = 1.72; 95% CI, 1.20-2.47), the MR of diabetes (quintile 5; IRR = 1.49; 95% CI, 1.15-1.93), proportion of indigenous population (quintile 4; IRR = 1.51; 95% CI, 1.20-1.91), proportion of economically active population (quintile 5; IRR = 1.59; 95% CI, 1.09-1.32), and population density (quintile 5; IRR = 2.5; 95% CI, 1.78-3.51). Factors inversely associated with lower mortality from COVID-19 at the municipality level were the hypertension prevalence (quintile 5; IRR = 0.39; 95% CI, 0.29-0.52) and characteristics of marginalized populations, such as illiteracy (quintile 5; IRR = 0.62; 95% CI, 0.40-0.95), houses without sewage drainage (quintile 5; IRR =0.71; 95% CI, 0.51-0.90), houses with dirt floors (quintile 5; IRR= 0.68; 95%CI, 0.47-1.00), proportion of population without health-care insurance (quintile 5; IRR = 0.66; 95% CI, 0.45-0.96) (Table 2).

**Table 2.** Municipal- and state-level factors associated with higher cumulative incidence, mortality, and case fatality from COVID-19 in symptomatic cases in Mexico as of June 20, 2020

		COVID-19 cumulative incidence of confirmed cases^§^		COVID-19 MR^£^		COVID-19 case fatality rate^¥^	
	*n*	175 148		20 773		175 148	
	Q	IRR (95% CI)	*P*-Value	IRR (95% CI)	*P*-Value	IRR (95%CI)	*P*-Value
Proportion of population aged 60 y or older^a^	1	Ref.		Ref.		Ref.	
	2	1.11 (0.98, 1.26)	0.108	1.07 (0.92, 1.24)	0.399	1.1 (0.77, 1.57)	0.606
	3	1.18 (1.03, 1.36)	0.016	1.15 (0.96, 1.37)	0.119	1.59 (1.08, 2.34)	0.018
	4	1.06 (0.91, 1.23)	0.472	1.11 (0.91, 1.36)	0.296	1.50 (0.98, 2.29)	0.061
	5	1.07 (0.88, 1.3)	0.490	1.25 (0.93, 1.68)	0.131	1.57 (0.94, 2.62)	0.085
Proportion of males^a^	1	Ref.		Ref.			
	2	1 (0.87, 1.16)	0.987	1.01 (0.84, 1.22)	0.919	0.59 (0.4, 0.85)	0.005
	3	0.91 (0.78, 1.07)	0.250	0.96 (0.79, 1.17)	0.697	0.7 (0.47, 1.04)	0.08
	4	0.94 (0.8, 1.1)	0.422	1.00 (0.81, 1.23)	0.990	0.68 (0.45, 1.05)	0.085
	5	0.92 (0.76, 1.11)	0.388	1.13 (0.88, 1.46)	0.341	0.63 (0.39, 1.03)	0.064
State-level prevalence of diabetes in adults aged 20 y or older^b^	1	Ref.		Ref.		Ref.	
	2	1.05 (0.9, 1.22)	0.562	1.30 (1.08, 1.58)	0.007	2.35 (1.54, 3.58)	<0.001
	3	1.27 (1.06, 1.52)	0.009	2.01 (1.60, 2.54)	<0.001	1.52 (0.92, 2.53)	0.105
	4	1.22 (0.98, 1.52)	0.07	2.60 (1.97, 3.43)	<0.001	1.24 (0.67, 2.27)	0.491
	5	1.39 (1.13, 1.70)	0.001	2.14 (1.66, 2.75)	<0.001	2.11 (1.19, 3.74)	0.010
State-level prevalence of obesity in adults aged 20 y or older^b^	1	Ref.		Ref.		Ref.	
	2	0.77 (0.66, 0.91)	0.001	0.71 (0.58, 0.88)	0.001	1.20 (0.77, 1.86)	0.422
	3	0.85 (0.71, 1.02)	0.076	0.9 (0.71, 1.13)	0.372	1.29 (0.76, 2.20)	0.352
	4	0.72 (0.58, 0.9)	0.004	0.61 (0.46, 0.8)	0.001	1.10 (0.55, 2.19)	0.793
	5	2.37 (1.8, 3.12)	<0.001	1.93 (1.37, 2.71)	<0.001	2.37 (1.06, 5.29)	0.035
State-level prevalence of hypertension in adults aged 20 y or older^b^	1	Ref.		Ref.		Ref.	
	2	0.9 (0.73, 1.11)	0.317	0.59 (0.45, 0.78)	<0.001	1.01 (0.54, 1.9)	0.980
	3	0.84 (0.7, 1.01)	0.058	0.58 (0.46, 0.74)	<0.001	0.61 (0.36, 1.02)	0.060
	4	1.14 (0.92, 1.39)	0.228	0.55 (0.42, 0.71)	<0.001	0.55 (0.29, 1.02)	0.059
	5	0.62 (0.5, 0.76)	<0.001	0.40 (0.31, 0.53)	<0.001	0.99 (0.56, 1.76)	0.980
Crude MR of diabetes in adults 20 y or older^c^	1	Ref.		Ref.		Ref.	
	2	1.05 (0.91, 1.22)	0.507	1.17 (0.94, 1.45)	0.169	1.04 (0.71, 1.52)	0.843
	3	1.05 (0.91, 1.23)	0.495	1.34 (1.07, 1.66)	0.009	0.78 (0.53, 1.15)	0.207
	4	1.16 (1.00, 1.36)	0.055	1.54 (1.23, 1.94)	<0.001	0.79 (0.54, 1.17)	0.245
	5	1.23 (1.04, 1.45)	0.014	1.58 (1.24, 2.01)	<0.001	0.73 (0.49, 1.09)	0.126
Crude MR of hypertension in adults 20 y or older^c^	1	Ref.		Ref.		Ref.	
	3	1 (0.87, 1.14)	0.945	0.94 (0.8, 1.11)	0.486	0.52 (0.36, 0.75)	<0.001
	4	1.02 (0.91, 1.15)	0.704	1.02 (0.87, 1.2)	0.816	0.81 (0.59, 1.12)	0.204
	5	0.93 (0.83, 1.06)	0.274	1.03 (0.85, 1.25)	0.738	1.02 (0.76, 1.39)	0.877
Crude MR of cerebrovascular disease in adults 20 y or older^c^	1	Ref.		Ref.		Ref.	
	2	0.91 (0.76, 1.08)	0.279	0.95 (0.75, 1.19)	0.632	0.99 (0.61, 1.6)	0.967
	3	0.98 (0.85, 1.13)	0.796	1.01 (0.82, 1.24)	0.921	0.89 (0.61, 1.31)	0.562
	4	0.96 (0.83, 1.09)	0.509	1.04 (0.85, 1.27)	0.712	1.05 (0.73, 1.51)	0.802
	5	0.91 (0.79, 1.05)	0.185	0.93 (0.74, 1.17)	0.536	1.04 (0.75, 1.46)	0.806
Proportion of the population in extreme poverty	1	Ref.		Ref.		Ref.	
	2	1.07 (0.92, 1.25)	0.361	1.04 (0.86, 1.27)	0.655	1.25 (0.81, 1.93)	0.312
	3	1.11 (0.9, 1.35)	0.324	1.10 (0.84, 1.44)	0.487	1.44 (0.81, 2.55)	0.216
	4	1.08 (0.85, 1.39)	0.523	1.21 (0.86, 1.70)	0.274	1.91 (0.95, 3.83)	0.069
	5	0.81 (0.59, 1.11)	0.192	1.00 (0.63, 1.60)	0.986	3.17 (1.37, 7.33)	0.007
Proportion of illiterate population aged 5 y or older^e^	1	Ref.		Ref.		Ref.	
	2	0.72 (0.62, 0.83)	<0.001	0.75 (0.63, 0.9)	0.002	0.88 (0.6, 1.3)	0.529
	3	0.63 (0.53, 0.77)	<0.001	0.68 (0.53, 0.87)	0.002	0.75 (0.46, 1.22)	0.243
	4	0.55 (0.44, 0.68)	<0.001	0.62 (0.46, 0.84)	0.002	1.45 (0.82, 2.55)	0.199
	5	0.50 (0.38, 0.65)	<0.001	0.62 (0.42, 0.92)	0.018	1.11 (0.57, 2.16)	0.767
Proportion of indigenous population aged 5 y or older^e^	1	Ref.		Ref.		Ref.	
	2	0.91 (0.8, 1.04)	0.166	1.43 (1.2, 1.71)	<0.001	1.01 (0.72, 1.43)	0.934
	3	0.94 (0.81, 1.08)	0.366	1.68 (1.38, 2.03)	<0.001	0.84 (0.58, 1.21)	0.356
	4	0.77 (0.66, 0.9)	0.001	1.39 (1.13, 1.73)	0.002	1.13 (0.75, 1.7)	0.544
	5	0.70 (0.57, 0.86)	0.001	1.18 (0.87, 1.61)	0.297	0.79 (0.48, 1.32)	0.369
Proportion of population economically active aged 12 y or older^d^	1	Ref.		Ref.		Ref.	
	2	1.18 (1.00, 1.40)	0.048	1.22 (0.92, 1.63)	0.165	1.41 (0.97, 2.06)	0.074
	3	1.38 (1.15, 1.66)	0.001	1.29 (0.95, 1.74)	0.102	1.20 (0.78, 1.86)	0.410
	4	1.42 (1.16, 1.74)	0.001	1.35 (0.97, 1.86)	0.071	1.83 (1.11, 3.01)	0.018
	5	1.61 (1.28, 2.04)	<0.001	1.50 (1.06, 2.14)	0.024	1.67 (0.94, 2.94)	0.078
Rate of economic units that operate essential activities during COVID-19 outbreak^d^	1	Ref.		Ref.		Ref.	
	2	1.41 (1.22, 1.62)	<0.001	1.33 (1.08, 1.64)	0.007	1.67 (1.17, 2.38)	0.005
	3	1.34 (1.15, 1.56)	<0.001	1.21 (0.97, 1.50)	0.094	1.58 (1.08, 2.31)	0.019
	4	1.60 (1.36, 1.88)	<0.001	1.54 (1.23, 1.93)	<0.001	1.07 (0.72, 1.59)	0.730
	5	1.48 (1.24, 1.77)	<0.001	1.29 (1.01, 1.66)	0.043	1.40 (0.91, 2.16)	0.124
Proportion of houses without water service inside the domicile^d^	1	Ref.		Ref.		Ref.	
	2	0.81 (0.71, 0.93)	0.002	1.58 (1.21, 2.07)	0.057	1.02 (0.72, 1.45)	0.918
	3	0.93 (0.81, 1.07)	0.324	1.67 (1.27, 2.19)	0.910	0.93 (0.63, 1.36)	0.702
	4	1.1 (0.94, 1.29)	0.219	2.28 (1.71, 3.04)	0.671	0.84 (0.56, 1.25)	0.377
	5	1.27 (1.07, 1.51)	0.006	2.72 (1.99, 3.73)	0.063	1.10 (0.70, 1.73)	0.675
Proportion of houses without sewage drainage service^d^	1	Ref.		Ref.		Ref.	
	2	1.01 (0.88, 1.15)	0.934	0.85 (0.72, 1)	0.365	1.04 (0.71, 1.52)	0.846
	3	1.03 (0.88, 1.20)	0.72	1.01 (0.84, 1.21)	0.567	1.11 (0.73, 1.67)	0.624
	4	0.95 (0.8, 1.14)	0.601	1.04 (0.86, 1.27)	0.760	1.09 (0.68, 1.74)	0.720
	5	0.80 (0.65, 1.00)	0.047	1.24 (0.99, 1.55)	0.065	0.62 (0.36, 1.04)	0.072
Proportion of houses with dirt floor^d^	1	Ref.		Ref.		Ref.	
	2	1.12 (0.98, 1.29)	0.093	0.93 (0.78, 1.09)	0.037	1.69 (1.15, 2.48)	0.008
	3	1.25 (1.05, 1.48)	0.011	0.94 (0.77, 1.16)	0.094	0.99 (0.62, 1.57)	0.959
	4	1.03 (0.84, 1.26)	0.774	0.96 (0.76, 1.22)	0.798	1.01 (0.61, 1.68)	0.969
	5	1.09 (0.85, 1.39)	0.492	0.75 (0.55, 1.02)	0.204	0.61 (0.32, 1.17)	0.139
Rate of hospitals/population^a^	1	Ref.		Ref.			
	2	1.25 (1.1, 1.41)	0.001	1.21 (1.01, 1.44)	0.005	0.60 (0.42, 0.86)	0.005
	3	1.16 (1.03, 1.3)	0.015	1.21 (0.97, 1.52)	0.482	0.76 (0.56, 1.04)	0.087
Rate of units of health-care services/population^a^	1	Ref.		Ref.		Ref.	
	2	0.97 (0.85, 1.1)	0.592	0.97 (0.74, 1.27)	0.598	1.11 (0.78, 1.57)	0.579
	3	0.9 (0.77, 1.04)	0.163	0.8 (0.56, 1.13)	0.673	1.05 (0.7, 1.59)	0.802
	4	1.15 (0.97, 1.37)	0.112	1.25 (1.07, 1.45)	0.599	1.27 (0.79, 2.04)	0.33
	5	1.12 (0.89, 1.4)	0.327	1.06 (0.9, 1.24)	0.866	2.47 (1.37, 4.46)	0.003
Rate of social assistance medical units/population^a^	1	Ref.		Ref.		Ref.	
	4	1.08 (0.91, 1.27)	0.373	1.04 (0.9, 1.21)	0.526	0.35 (0.22, 0.56)	<0.001
	5	1.35 (1.2, 1.51)	<0.001	0.96 (0.79, 1.16)	0.074	0.78 (0.58, 1.04)	0.089
Population density^c^	1	Ref.					
	2	1.20 (1.01, 1.43)	0.035	1.07 (0.84, 1.37)	0.001	1.14 (0.76, 1.72)	0.535
	3	1.23 (1.02, 1.47)	0.027	1.03 (0.72, 1.49)	<0.001	1.42 (0.90, 2.22)	0.131
	4	1.70 (1.4, 2.07)	<0.001	1.06 (0.89, 1.26)	<0.001	1.31 (0.78, 2.19)	0.304
	5	2.08 (1.67, 2.6)	<0.001	1.14 (0.99, 1.31)	<0.001	1.57 (0.86, 2.87)	0.138
Proportion of population without health-care insurance^f^	1	Ref.		Ref.		Ref.	
	2	0.72 (0.61, 0.84)	<0.001	0.71 (0.59, 0.86)	0.067	0.92 (0.6, 1.4)	0.698
	3	0.65 (0.53, 0.80)	<0.001	0.60 (0.47, 0.77)	0.006	0.86 (0.52, 1.44)	0.577
	4	0.68 (0.54, 0.86)	0.001	0.70 (0.52, 0.94)	0.011	0.98 (0.55, 1.76)	0.956
	5	0.77 (0.59, 1.00)	0.053	0.64 (0.45, 0.92)	0.031	1.02 (0.53, 1.96)	0.955
Proportion of population affiliated to the Mexican Social Security Institute (IMSS)^d^	1	Ref.		Ref.		Ref.	
	2	0.95 (0.80, 1.13)	0.578	0.86 (0.66, 1.13)	0.288	1.54 (1.01, 2.35)	0.047
	3	0.95 (0.79, 1.15)	0.617	1.02 (0.76, 1.38)	0.876	1.67 (1.02, 2.72)	0.040
	4	0.87 (0.70, 1.07)	0.178	0.97 (0.70, 1.35)	0.864	1.50 (0.86, 2.62)	0.158
	5	0.80 (0.62, 1.04)	0.090	0.83 (0.57, 1.21)	0.327	1.02 (0.52, 1.98)	0.961
Proportion of population with private health insurance^d^	1	Ref.		Ref.		Ref.	
	2	1.20 (1.01, 1.42)	0.037	1.05 (0.8, 1.38)	0.729	1.20 (0.81, 1.76)	0.364
	3	1.37 (1.15, 1.63)	0.001	1.16 (0.88, 1.53)	0.303	0.81 (0.53, 1.25)	0.345
	4	1.17 (0.97, 1.41)	0.098	0.98 (0.73, 1.31)	0.871	0.84 (0.54, 1.31)	0.443
	5	1.28 (1.05, 1.56)	0.016	1.01 (0.75, 1.37)	0.938	0.85 (0.52, 1.38)	0.511

Abbreviation: Q, quintiles.

§ COVID-19 symptomatic cases/population.

£ COVID-19 deaths in symptomatic cases/population.

¥ COVID-19 deaths in symptomatic cases/symptomatic cases.

a Municipality data of 2020.

b State data of 2018.

c Municipality data of 2018.

d Municipality data of 2015.

e Municipality data of 2010.

f Municipality data of 2019.

The sample includes 20,773 from the 175,148 accumulated confirmed cases with complete information of June 20, 2020.

^1^SSA: Ministry of Health Hospitals.

^2^IMSS: Mexican Social Security Institute.

^3^ISSSTE: The Mexican Civil Service Social Security and Services Institute. References categories were <40 y old, females, and not having the condition.

**Figure 1.**
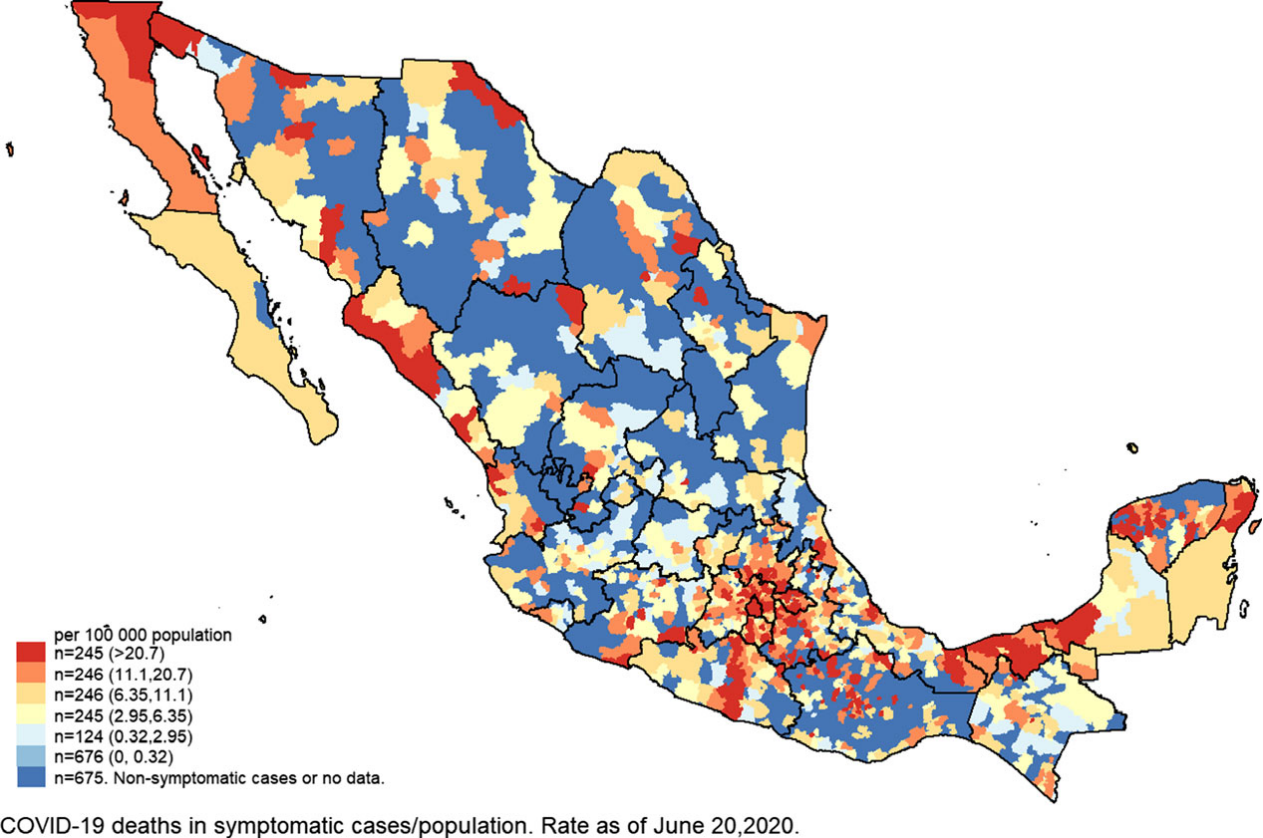
Crude MR of symptomatic cases of COVID-19 in Mexico as of June 20, 2020.

**Figure 2.**
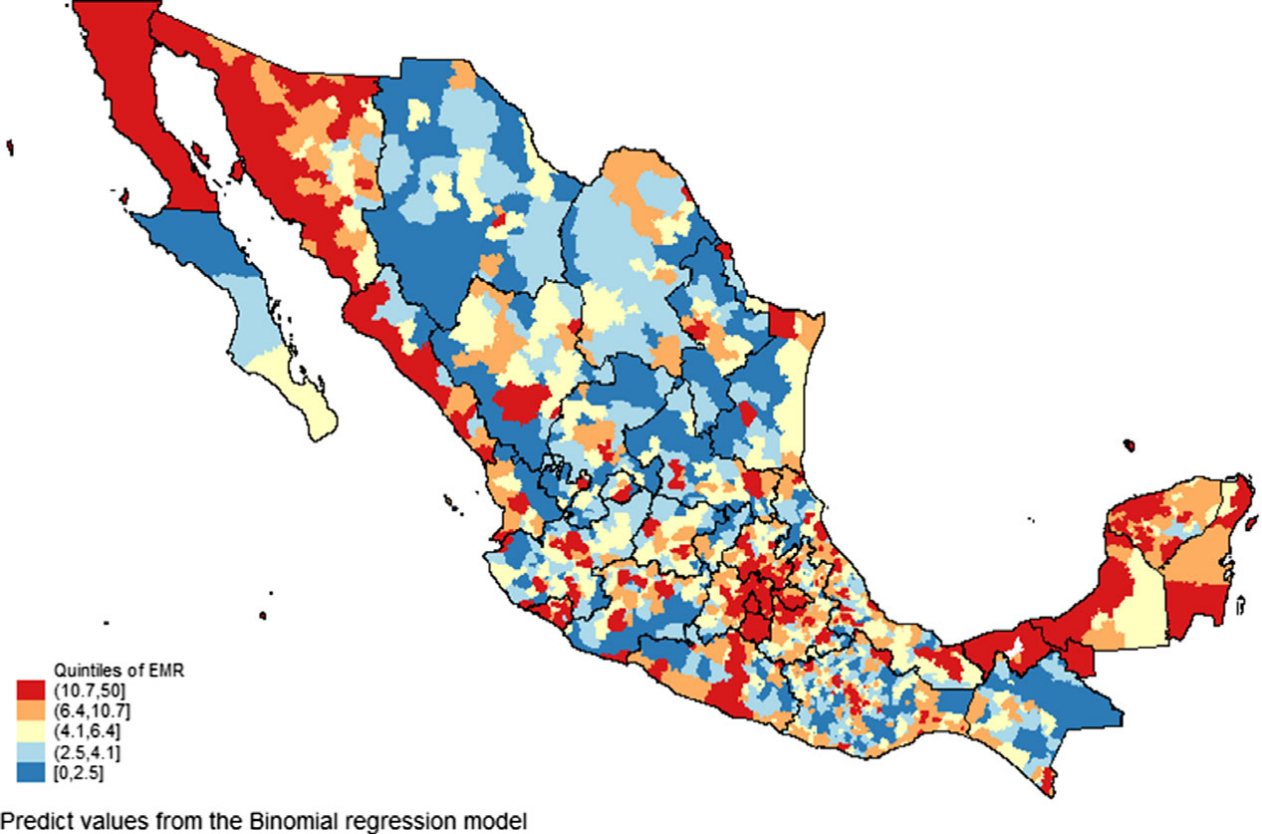
EMR from COVID-19 according to municipal factors studied.

Municipality-level factors associated with high cumulative incidence rate of COVID-19 were similar to those associated to high mortality, except by the proportion of indigenous population (quintile 5; IRR = 0.70; 95% CI, 0.57-0.86) and the proportion of houses without sewage drainage (quintile 5; IRR = 0.80; 95% CI, 0.65-1.00), which were inversely associated (Table 2). Finally, municipality-level factors associated with higher case fatality from COVID-19 were as well the prevalence of diabetes (quintile 5; IRR = 2.11; 95% CI, 1.19-3.74), obesity prevalence (quintile 5; IRR = 2.37; 95% CI, 1.06-5.29), extreme poverty (quintile 5; IRR = 3.17; 95% CI, 1.37-7.33), and rate of social assistance medical units (quintile 5; IRR = 2.47; 95% CI, 1.37-4.46). Quintiles of crude mortality and expected MRs from COVID-19 at the municipality level are shown in Figures 1 and 2, respectively.

We identified 1351 municipality levels without COVID-19 deaths reported in symptomatic cases, which according to its characteristics, 202 had high and 82 very high EMRs from COVID-19 (quintile 4; mean = 8.0; 95% CI, 5.7-11.2; and quintile 5; mean = 13.8; 95% CI, 9.97-19.1, respectively). Supplementary Table 1 lists the name and characteristics of the municipality level with high expected MR COVID-19, based on the significant risk factors identified in the binomial regression model (Table 2). It is observed that many municipality levels of the states of Oaxaca (*n* = 68), Yucatán (*n* = 60), Sonora (*n* = 33), Veracruz (*n* = 25), Puebla (*n* = 24), and Michoacán (*n* = 18) had the highest expected MRs (quintile 4; mean = 8.01; 95% CI, 7.85-8.17; and quintile 5; mean = 13.7; 95% CI, 13.1-14.4). It was observed that 43 municipality levels with high expected mortality from COVID-19 are Hope Municipality- levels. Case-fatality rates from COVID-19 in symptomatic cases are shown in Supplementary Map 1. It can be observed that some municipality levels of the States of Oaxaca, Chihuahua, and Guerrero had case-fatality rates from COVID-19 higher than 80%.

At the individual level, we also explored characteristics of the confirmed symptomatic cases that were associated with a higher lethality of COVID-19 to determine if the risk factors observed in other countries remain in the Mexican context (Supplementary Table 2). In concordance with the municipality-level results, diabetes, belonging to an indigenous ethnic group, and obesity were associated with higher risk of mortality due to coronavirus. Other individual risk factors of lethality cases were age 40 or older, male sex, hypertension, CKD, COPD, and immunosuppression. In addition, having received medical care in private health institutions was associated with a lower lethality from COVID-19 compared with those cared for at public health services. As for individuals who sought medical attention between 6 and 14 d after the onset of symptoms, they had a higher risk of dying compared with those who received medical care between 0 and 5 d after the onset of symptoms.

In Supplementary Table 3, we illustrate the municipality-level factors associated to the cumulative incidence and lethality rates as of June 20, 2020, disaggregated by month. We found that municipality-level factors association with cumulative incidence and MR from COVID-19 varied within the course of the pandemic in Mexico.

## Discussion

Municipality-level factors found to be associated with higher risk of mortality from COVID-19 were the population density, the prevalence of diabetes and obesity, the MR of diabetes, the proportion of indigenous population, of economically active population, and the rate of economic units that operate essential activities during the COVID-19 outbreak.

First, a high proportion of the population economically active may reflect less confinement and increased mobility, while higher population density may be related to less social distancing and high connectivity. Second, the high MR of persons with diabetes means inadequate control of glucose levels, which has a negative impact on the immune and cardiovascular systems, both crucial in the body’s response to COVID-19.^26^ Finally, factors included lower access to care, quality of medical care, or weaker local health-care systems.^27^

At the individual level, hypertension was associated with higher lethality from COVID-19. At the municipality level, the MR of hypertension was not associated with high mortality from COVID-19, and within states with the highest prevalence of hypertension, they had lower MR from COVID-19. This result could be explained by the fact that the prevalence used in our model came from adults already diagnosed with hypertension, which according to previous surveys represented approximately 60% of the total cases of hypertensive population and are those with access to health-care services and antihypertensive medication.^28^ Nevertheless, unknown and unobserved variables, before the demand of health-care services might be reflecting an inverse association between hypertension and COVID-19 mortality.

It is important to note that the cumulative incidence of cases in indigenous populations was low; however, the risk of mortality from coronavirus was higher than in municipalities with a lower proportion of indigenous population. These findings could indicate a lower access to health-care services and testing, combined with a dynamic where the epidemic migrates further into more marginalized areas with a higher proportion of indigenous population. Other possible reason that could explain higher mortality could be due to a delay in the time of seeking for diagnosis and medical treatment. Other countries have found that many mechanisms underlying the higher lethality of COVID-19 in ethnic minorities are related to marginalization conditions, such as locality of residence, work conditions, and health inequalities.^29,30^

Marginalized conditions, such as no sewage service or dirt floors, were inversely associated with mortality from COVID-19. Communities with these household characteristics are mostly remote and with low connectivity, which could be protective from SARS-CoV-2 exposure. Notwithstanding, marginalized and remote communities could also have lower access to both testing for SARS-CoV-2 and health care, which could lead to underreporting of cases and deaths. Special screening strategies and tracing and isolation of contacts within these vulnerable communities are essential to favor the prevention of deaths from COVID-19.^31^.

In May 2020, the Mexican MoH published a list of 324 *Municipios de la Esperanza* (“Hope Municipalities”), which in the last 28 d before May 16 did not have any confirmed cases of COVID-19 and were not adjacent to municipalities with confirmed cases. This list was a guide for state governors to restart nonessential activities on May 18, 2020.^32^ However, according to our results, 16% of these municipality levels are at high risk of having worse outcomes in the event of an outbreak and, as of June 20, 2020, a total of 389 symptomatic cases and 29 deaths due to COVID-19 were confirmed in these municipalities.

The identification of municipalities with a high burden of risk factors of severe illness or death from COVID-19 is critical to establish the most convenient health policies at the municipality level. Many of these municipalities are in locations susceptible to the onset of an outbreak as the pandemic evolves in Mexico. Municipalities with no confirmed cases yet, but with populations at risk of becoming seriously ill from COVID-19, could have a greater burden of the disease in the upcoming months if contingency measures and mobility restrictions are eased to soon. A targeted approach will be crucial to prevent or control the onset of new outbreaks within these municipalities, including testing, isolation, and contact tracing, as well as more general measures according to the 3-stage traffic light system that was established in Mexico to reactivate the economy and reduce contingency measures.^12^

In addition to the municipality-level factors associated with the cumulative incidence and mortality from COVID-19, we identified that municipalities with conditions of extreme poverty had higher case-fatality rates from COVID-19. For example, Oaxaca, Chihuahua, and Guerrero are states in which some municipalities had case-fatality rates higher than 80%. This finding suggests a low availability of tests in this communities.

As per the limitations within our study, the precision of the estimations depends on the quality of the databases, for example; the aging of our data for some variables is up to 10 y, which may not reflect heterogeneous changes within municipalities by 2020. Nevertheless, data from previous years are still relevant to the following years, and they were useful to find expected associations. For obesity, diabetes, and hypertension prevalence, the information from the ENSANUT-2018 are representative at the state level, reducing the variability within municipalities. However, for the rest of the variables, such as MRs of diabetes and hypertension, information was disaggregated at the municipality. Finally, the ecological design of our study prevented us to from establishing causal associations.

To our knowledge, this is the first study that estimated MRs from COVID-19 by using the burden of related comorbidities and sociodemographic characteristics to identify municipalities at risk of high MRs of coronavirus in Mexico. Our findings could contribute to the national strategic preparedness and response plans toward a “new normality”^33^ by informing, at a municipality-level, factors to consider in the decision-making process and public health interventions to minimize the negative impact of COVID-19 on the health and livelihoods of the most at-risk communities.

Based on our results, we considered this is a good moment to modify the current epidemiological surveillance strategy for confirming COVID-19 positive cases and deaths in populations that might be under-reported by the Sentinel surveillance approach. For instance, indigenous communities and communities with extreme poverty could be affected not only by the risks of COVID-19 afflicting the health of the population, but also by increasing food insecurity, domestic violence, disrupting the routine care of chronic diseases, or the economic repercussions this might bring. It is critical to count on data on the impact of COVID-19 among these populations to identify, prioritize, and address the needs of these vulnerable populations.

## Conclusions

Using small area demographic characteristics and burden of comorbidities on COVID-19 is useful to identify locations at risk of COVID-19 mortality. In Mexico, municipality-level risk factors associated with high MRs from COVID-19 were a high proportion of the population economically active, high population density, high proportion of indigenous population, and high diabetes mortality. Based on their characteristics, many of the municipalities that have not experienced high mortality yet are prone to do so as the epidemic curve progresses. We, therefore, warn against overconfidence and premature easing of mobility restrictions and other contingency measures. Local governments ought to reinforce local strategies to prevent outbreaks in vulnerable communities to COVID-19.

## Supplementary material

For supplementary material accompanying this paper visit https://doi.org/10.1017/dmp.2020.485. click here to view supplementary material

## References

[1] Ministry of Health of Mexico. Coronavirus technical news release. February 28, 2020. https://www.gob.mx/cms/uploads/attachment/file/538453/Comunicado_Tecnico_Diario_COVID-19_2020.02.28.pdf. Accessed May 29, 2020.

[2] Ministry of Health of Mexico. Preparedness and response actions in Mexico. February 27, 2020. https://www.gob.mx/salud/prensa/076-covid-19-acciones-de-preparacion-y-respuesta-en-mexico. Accessed May 29, 2020.

[3] Ministry of Health of Mexico. Jornada Nacional de Sana Distancia. March 23, 2020. https://www.gob.mx/cms/uploads/attachment/file/541687/Jornada_Nacional_de_Sana_Distancia.pdf. Accessed May 29, 2020.

[4] Ministry of Health of Mexico. Open data – Dirección General de Epidemiología. Updated June 21, 2020. https://coronavirus.gob.mx/datos/. Accessed June 22, 2020.

[5] Zheng Z , Peng F , Xu B , et al. Risk factors of critical & mortal COVID-19 cases: a systematic literature review and meta-analysis. J Infect. 2020;81(2):e16–e25. doi: 10.1016/j.jinf.2020.04.021 PMC7177098 32335169

[6] Centers for Disease Control and Prevention. Groups at higher risk for severe illness. May 14, 2020. https://www.cdc.gov/coronavirus/2019-ncov/need-extra-precautions/groups-at-higher-risk.html. Accessed May 29, 2020.

[7] National Institute of Statistics and Geography and National Institute of Public Health. National Survey on Health and Nutrition 2018 (ENSANUT 2018). Results report. https://ensanut.insp.mx/encuestas/ensanut2018/doctos/informes/ensanut_2018_presentacion_resultados.pdf. Accessed May 29, 2020.

[8] Popovich N , Singhvi A , Conlen M. Where chronic health conditions and coronavirus could collide. New York Times. May 18, 2020. https://www.nytimes.com/interactive/2020/05/18/us/coronavirus-underlying-conditions.html. Accessed May 22, 2020.

[9] Chin T , Kahn R , Li R , et al. U.S. county-level characteristics to inform equitable COVID-19 response. Medivix preprint. April 11, 2020. 10.1101/2020.04.08.20058248 https://www.medrxiv.org/content/10.1101/2020.04.08.20058248v1.full.pdf. Accessed May 29, 2020.

[10] Tiana T , Zhanga J , Liyuan Hua L , et al. Risk factors associated with mortality of COVID-19 in 2692 counties of the United States. Medivix preprint. June 2, 2020. 10.1101/2020.05.18.20105544 https://www.medrxiv.org/content/10.1101/2020.05.18.20105544v3.full.pdf. Accessed May 29, 2020.

[11] National Institute of Statistics and Geography. Geographical areas. Mexico. https://en.www.inegi.org.mx/datos/default.html?t=0150#Geographical_areas. Accessed May 29, 2020.

[12] ACUERDO por el que se establece una estrategia para la reapertura de las actividades sociales, educativas y económicas, así como un sistema de semáforo por regiones para evaluar semanalmente el riesgo epidemiológico relacionado con la reapertura de actividades en cada entidad federativa, así como se establecen acciones extraordinarias. Diario Oficial de la Federación. May 15, 2020. https://dof.gob.mx/nota_detalle.php?codigo=5593313&fecha=14/05/2020. Accessed May 29, 2020.

[13] Pan American Health Organization. Operational guidelines for Sentinel Severe Acute Respiratory Infection (SARI) surveillance. September 2014. https://www.paho.org/revelac-i/wp-content/uploads/2015/10/2015-cha-operational-guidelines-sentinel-sari.pdf. Accessed May 29, 2020.

[14] Ministry of Health. COVID-19 guidelines for patient care - Lineamiento para la atención de pacientes por COVID-19. February 14, 2020. https://drive.google.com/file/d/1vge89Fuz_9RsgKk77XrpyG2RYW7NAGFP/view. Accessed May 29, 2020.

[15] Institute of Epidemiological Diagnosis and Reference (InDRE). Benchmarking process for the identification of the SARS-CoV-2 virus, causal agent of COVID-19. Updated June 18, 2020. https://www.gob.mx/salud/documentos/coronavirus-covid-19-240014?state=published. Accessed June 22, 2020.

[16] National Institute of Statistics and Geography. National geostatistical framework. Mexico. https://en.www.inegi.org.mx/temas/mg/default.html# Downloads. Accessed June 20, 2020.

[17] National Institute of Statistics and Geography. Census 2010. Mexico. https://en.www.inegi.org.mx/programas/ccpv/2010/. Accessed May 29, 2020.

[18] National Institute of Statistics and Geography. Intercensal Survey 2015. Mexico. Available at: http://en.www.inegi.org.mx/programas/intercensal/2015/. Accessed May 29, 2020.

[19] National Institute of Statistics and Geography. Percentage of the population affiliated to popular insurance. Mexico. April 30, 2019. https://datos.gob.mx/busca/dataset/porcentaje-de-la-poblacion-afiliada-al-seguro-popular-derecho-a-la-salud-recepcion-del-derecho. Accessed May 29, 2020.

[20] National Institute of Statistics and Geography. National directory of economic units. June 2020. https://www.inegi.org.mx/app/descarga/?ti=6. Accessed June 20, 2020.

[21] National Council for the Evaluation of Social Development Policy. Medición de la pobreza. Pobreza a nivel municipio 2010 y 2015. https://www.coneval.org.mx/Medicion/Paginas/Pobreza-municipal.aspx. Accessed June 20, 2020.

[22] Department of Epidemiological Surveillance of the Ministry of Health. Open data. Updated June 21, 2020. https://www.gob.mx/salud/documentos/datos-abiertos-152127. Accessed June 21, 2020.

[23] Mexican Government. Unique Key of Health Establishments (CLUES) Catalog. Mexico. June 1, 2020. http://www.dgis.salud.gob.mx/contenidos/intercambio/clues_gobmx.html. Accessed June 10, 2020.

[24] General Secretariat of the National Population Council. CONAPO. Projections of the population of Mexico and of the Federal Entities, 2016-2050. September 17, 2018. https://datos.gob.mx/busca/dataset/proyecciones-de-la-poblacion-de-mexico-y-de-las-entidades-federativas-2016-2050. Accessed May 29, 2020.

[25] National Institute of Statistics and Geography. Mexican National Health and Nutrition Survey 2018 (ENSANUT 2018). https://www.inegi.org.mx/programas/ensanut/2018/. Accessed May 29, 2020.

[26] Bornstein SF , Rubino F , Khunti K , et al. Practical recommendations for the management of diabetes in patients with COVID-19. Lancet Diabetes Endocrinol. 2020;8(6);546–550. doi: 10.1016/S2213-8587(20)30152-2 32334646 PMC7180013

[27] Kruk ME , Gage AD , Joseph NT , et al. Mortality due to low-quality health systems in the universal health coverage era: a systematic analysis of amenable deaths in 137 countries. Lancet. 2018;392:2203–2212. doi: 10.1016/S0140-6736(18)31668-4 30195398 PMC6238021

[28] Campos-Nonato I , Hernández-Barrera L , Pedroza-Tobías A , et al. Hypertension in Mexican adults: prevalence, diagnosis and type of treatment. Ensanut MC 2016. Salud Publica Mex. 2018;60:233–243. doi: 10.21149/8813 29746740

[29] Centers for Disease Control and Prevention. COVID-19 in racial and ethnic minority groups. June 4, 2020. https://www.cdc.gov/coronavirus/2019-ncov/need-extra-precautions/racial-ethnic-minorities.html. Accessed June 20, 2020.

[30] Pan D , Sze S , Minhas JS , et al. The impact of ethnicity on clinical outcomes in COVID-19: a systematic review. EClinicalMedicine. 2020;23:100404. doi: 10.1016/j.eclinm.2020.100404 32632416 PMC7267805

[31] Martinez-Juarez LA , Sedas AC , Orcuttd M , et al. Governments and international institutions should urgently attend to the unjust disparities that COVID-19 is exposing and causing. EClinicalMedicine. 2020;23:100376. doi: 10.1016/j.eclinm.2020.100376 32632411 PMC7272149

[32] Ministry of Health of Mexico. Hope municipalities. May 16, 2020. https://coronavirus.gob.mx/wp-content/uploads/2020/05/Municipios_Esperanza_16052020.pdf. Accessed May 29, 2020.

[33] Mexican Government. ACUERDO por el que se modifica el diverso por el que se establece una estrategia para la reapertura de las actividades sociales, educativas y económicas, así como un sistema de semáforo por regiones para evaluar semanalmente el riesgo epidemiológico relacionado con la reapertura de actividades en cada entidad federativa, así como se establecen acciones extraordinarias, publicado el 14 de mayo de 2020. May 15, 2020. https://www.dof.gob.mx/nota_detalle.php?codigo=5593411&fecha=15/05/2020. Accessed May 29, 2020.

