# Supplementary material for: Municipality-Level Predictors of COVID-19 Mortality in Mexico: A Cautionary Tale
Source: Disaster Med Public Health Prep. 2020 Dec 22:1–9. doi: 10.1017/dmp.2020.485 (PMC7985638; doi:10.1017/dmp.2020.485)
Supplement: Supplementary file 1 [file S1935789320004851sup001.docx]

| Supplementary Table 1. Expected mortality of COVID-19 in municipalities without COVID-19 deaths registered in symptomatic cases as for 20th of June, 2020. Characteristics of municipalities are displayed in quintiles of the distribution within 2457 municipalities of the country. | | | | | | | | | | | | |
| --- | --- | --- | --- | --- | --- | --- | --- | --- | --- | --- | --- | --- |
| State | Municipality | Expected COVID-10 mortality rate | Diabetes Mortality Rate | State diabetes prevalence | State Obesity Prevalence | Hypertension Mortality Rate | State hypertension prevalence | Indigenous population | Population density | Economically active | Hospitals | Hope Municipalities |
|  |  | Rate (95% CI) | Rate | % | % | Rate | % | % | Rate | % | Yes/No | Yes/No |
| Hidalgo | Tetepango | 27.6 (20.5, 37.3) | 263.9 | 12.9 | 31.2 | 12.6 | 17.9 | 0.3 | 258.7 | 46.6 | Yes | No |
| Yucatán | Muxupip | 21.6 (15.6, 29.7) | 189.1 | 10.7 | 44.4 | 0 | 21.7 | 41 | 39.6 | 47.4 | No | No |
| Yucatán | Akil | 19.5 (14, 27.2) | 85.2 | 10.7 | 44.4 | 0 | 21.7 | 63.2 | 147.9 | 49.4 | No | No |
| Hidalgo | Jaltocán | 18.8 (13.4, 26.5) | 190.6 | 12.9 | 31.2 | 0 | 17.9 | 88.8 | 307.8 | 41.2 | No | No |
| Morelos | Temoac | 17.5 (13, 23.5) | 174 | 12 | 33.5 | 9.7 | 17.5 | 0.6 | 427.2 | 49.3 | No | No |
| Yucatán | Sinanché | 17.4 (12, 25.1) | 215.2 | 10.7 | 44.4 | 43 | 21.7 | 28.4 | 23 | 43.2 | No | No |
| Oaxaca | Santa Inés Yatzeche | 17.3 (12.4, 24.1) | 311.5 | 10.5 | 30.4 | 0 | 17.1 | 94 | 395 | 32.6 | No | No |
| Yucatán | Tzucacab | 17.2 (12.2, 24.3) | 196.1 | 10.7 | 44.4 | 32.7 | 21.7 | 57.7 | 19.3 | 43.3 | No | No |
| Yucatán | Dzoncauich | 17.1 (11.9, 24.6) | 154.4 | 10.7 | 44.4 | 154.4 | 21.7 | 57.6 | 19.6 | 33.5 | No | Yes |
| Yucatán | Tahmek | 17.1 (11.9, 24.6) | 144 | 10.7 | 44.4 | 36 | 21.7 | 59.7 | 35.5 | 48.5 | No | No |
| Yucatán | Cansahcab | 17.1 (11.9, 24.6) | 143.9 | 10.7 | 44.4 | 57.6 | 21.7 | 25.4 | 35.6 | 44.5 | No | No |
| Yucatán | Dzidzantún | 17 (11.9, 24.3) | 146.2 | 10.7 | 44.4 | 0 | 21.7 | 8.7 | 39.4 | 48.4 | No | No |
| Yucatán | Mama | 16.8 (12.1, 23.4) | 142.1 | 10.7 | 44.4 | 0 | 21.7 | 76.1 | 32 | 49.2 | No | No |
| Oaxaca | San Mateo Yoloxochitlán | 16.8 (12.3, 23) | 190.1 | 10.5 | 30.4 | 0 | 17.1 | 77 | 507.6 | 40.3 | No | No |
| Yucatán | Sacalum | 16.3 (11.9, 22.3) | 121.5 | 10.7 | 44.4 | 0 | 21.7 | 61.2 | 24.2 | 51.3 | No | No |
| Oaxaca | Santa Lucía Ocotlán | 16.3 (12.1, 21.9) | 233.2 | 10.5 | 30.4 | 0 | 17.1 | 85.6 | 330.3 | 37.8 | No | Yes |
| Oaxaca | Chahuites | 16.3 (12.1, 21.9) | 212.1 | 10.5 | 30.4 | 0 | 17.1 | 5.6 | 503.4 | 47.9 | No | No |
| Yucatán | Tizimín | 16.1 (11.9, 21.9) | 108.1 | 10.7 | 44.4 | 4 | 21.7 | 42.6 | 20 | 46 | Yes | No |
| Yucatán | Telchac Puerto | 15.8 (11.3, 22.1) | 544.3 | 10.7 | 44.4 | 0 | 21.7 | 11.4 | 27.3 | 44.6 | No | No |
| Oaxaca | Santiago Apóstol | 15.8 (11.6, 21.6) | 183.6 | 10.5 | 30.4 | 0 | 17.1 | 75.4 | 226.9 | 40.7 | No | No |
| Guanajuato | Uriangato | 15.7 (11.9, 20.8) | 120.7 | 9.9 | 29.9 | 2.3 | 18.4 | 0.2 | 539.5 | 50.2 | Yes | No |
| México | Chiconcuac | 15.0 (11.2, 20.0) | 254.2 | 9 | 32.7 | 0 | 15.5 | 1.8 | 3734.4 | 53.1 | Yes | No |
| Yucatán | Yaxkukul | 14.8 (10.7, 20.5) | 269.5 | 10.7 | 44.4 | 0 | 21.7 | 11.7 | 62.8 | 50.1 | No | No |
| Guanajuato | Pueblo Nuevo | 14.7 (10.9, 20) | 197.8 | 9.9 | 29.9 | 12.4 | 18.4 | 0.2 | 198.3 | 40.3 | Yes | No |
| Coahuila | Allende | 14.7 (10.7, 20.3) | 163.3 | 12.4 | 38.6 | 13.1 | 22.4 | 0.2 | 90 | 46.5 | Yes | No |
| Veracruz | Oluta | 14.6 (10.7, 20) | 216.5 | 11.9 | 39.3 | 0 | 23.6 | 0.9 | 214.2 | 50.4 | Yes | No |
| Oaxaca | Ciénega de Zimatlán | 14.5 (10.4, 20.3) | 91.9 | 10.5 | 30.4 | 0 | 17.1 | 0.6 | 287.5 | 37.4 | No | No |
| Oaxaca | Asunción Ocotlán | 14.4 (10, 20.8) | 110.4 | 10.5 | 30.4 | 55.2 | 17.1 | 89.8 | 564.2 | 44.9 | No | Yes |
| Yucatán | Telchac Pueblo | 14.4 (9.7, 21.3) | 174.7 | 10.7 | 44.4 | 34.9 | 21.7 | 13.9 | 63.9 | 49.4 | No | No |
| Yucatán | Tepakán | 14.3 (9.9, 20.5) | 185 | 10.7 | 44.4 | 0 | 21.7 | 67.4 | 19.6 | 40.4 | No | No |
| Yucatán | Teya | 14.2 (9.8, 20.5) | 71.2 | 10.7 | 44.4 | 0 | 21.7 | 60.8 | 24.6 | 42.8 | No | No |
| Puebla | Rafael Lara Grajales | 14.1 (10.8, 18.5) | 292.6 | 9 | 32.7 | 0 | 15.4 | 0.3 | 3445 | 45.9 | Yes | No |
| Yucatán | Tetiz | 14.1 (10.2, 19.5) | 209.6 | 10.7 | 44.4 | 0 | 21.7 | 41 | 15.2 | 52.8 | No | No |
| SLP | Axtla de Terrazas | 13.9 (10, 19.5) | 110.3 | 10.8 | 33 | 17 | 19.2 | 55.2 | 196.9 | 40.5 | Yes | No |
| Puebla | Teteles de Avila Castillo | 13.7 (10.4, 18) | 152.6 | 9 | 32.7 | 0 | 15.4 | 17.2 | 600.1 | 46.2 | No | No |
| Oaxaca | Reyes Etla | 13.6 (9.8, 18.7) | 74.3 | 10.5 | 30.4 | 37.2 | 17.1 | 1.6 | 313.9 | 57.1 | No | No |
| Veracruz | Atoyac | 13.3 (9.6, 18.5) | 191.6 | 11.9 | 39.3 | 0 | 23.6 | 0.5 | 194.5 | 44.6 | Yes | No |
| México | Almoloya del Río | 13.3 (10.1, 17.5) | 232.4 | 9 | 32.7 | 0 | 15.5 | 0.6 | 1214.6 | 52.9 | No | No |
| Yucatán | Dzán | 13.2 (9.2, 19.1) | 56.8 | 10.7 | 44.4 | 28.4 | 21.7 | 73.3 | 67 | 48.1 | No | No |
| Puebla | Juan C. Bonilla | 13.1 (9.6, 17.8) | 190.4 | 9 | 32.7 | 7.6 | 15.4 | 1.1 | 953 | 47.7 | No | No |
| Tlaxcala | San Damián Texóloc | 13.1 (9.8, 17.3) | 202.2 | 9.3 | 32.1 | 0 | 13 | 0.2 | 545.4 | 49.5 | No | No |
| Puebla | Mixtla | 13 (9.8, 17.2) | 191.1 | 9 | 32.7 | 0 | 15.4 | 6 | 245.3 | 37.6 | No | No |
| Veracruz | Tomatlán | 12.9 (9.4, 17.6) | 163.9 | 11.9 | 39.3 | 41 | 23.6 | 0.3 | 365.8 | 47.1 | No | No |
| Chiapas | Tapilula | 12.9 (9.5, 17.4) | 227 | 7.8 | 29 | 0 | 16.2 | 8.8 | 302.6 | 44.7 | Yes | Yes |
| Oaxaca | San Agustín Yatareni | 12.7 (9.4, 17.2) | 171.8 | 10.5 | 30.4 | 34.4 | 17.1 | 14.5 | 677.2 | 48.5 | No | No |
| Puebla | Tlanepantla | 12.7 (9.6, 16.8) | 431.8 | 9 | 32.7 | 0 | 15.4 | 1.9 | 332.2 | 48.2 | No | No |
| Oaxaca | San Pedro Cajonos | 12.7 (8.9, 18.1) | 0 | 10.5 | 30.4 | 0 | 17.1 | 88.9 | 387.5 | 44.2 | No | Yes |
| Oaxaca | Natividad | 12.7 (8.9, 18.1) | 0 | 10.5 | 30.4 | 0 | 17.1 | 27.1 | 214.9 | 40.6 | No | Yes |
| Veracruz | Coscomatepec | 12.7 (9.4, 17) | 223.6 | 11.9 | 39.3 | 15.5 | 23.6 | 0.2 | 369.9 | 44.5 | Yes | No |
| Puebla | San Salvador Huixcolotla | 12.4 (9.2, 16.7) | 217.9 | 9 | 32.7 | 0 | 15.4 | 0.4 | 621.5 | 48.7 | Yes | No |
| Yucatán | Kinchil | 12.3 (8.7, 17.2) | 63.5 | 10.7 | 44.4 | 0 | 21.7 | 36.2 | 20.2 | 50.5 | No | No |
| Puebla | Tepatlaxco de Hidalgo | 12.1 (9, 16.3) | 427.1 | 9 | 32.7 | 18.6 | 15.4 | 11.5 | 284.9 | 46.9 | No | No |
| Puebla | General Felipe Ángeles | 12.1 (8.9, 16.4) | 209.4 | 9 | 32.7 | 40.3 | 15.4 | 0.6 | 224.1 | 40 | Yes | No |
| Yucatán | Yobaín | 12.1 (8.5, 17.2) | 61.7 | 10.7 | 44.4 | 0 | 21.7 | 21.3 | 16.9 | 44.1 | No | No |
| Veracruz | Zaragoza | 12.1 (8.7, 16.6) | 204 | 11.9 | 39.3 | 29.1 | 23.6 | 35.3 | 523.2 | 44.2 | No | No |
| Yucatán | Dzemul | 12 (8.4, 17.1) | 107.9 | 10.7 | 44.4 | 0 | 21.7 | 23.5 | 21 | 49.6 | No | No |
| Yucatán | Suma | 11.9 (8.2, 17.4) | 0 | 10.7 | 44.4 | 0 | 21.7 | 35.7 | 20 | 48 | No | No |
| Yucatán | Bokobá | 11.9 (8.3, 17) | 0 | 10.7 | 44.4 | 0 | 21.7 | 40.6 | 30.6 | 53 | No | No |
| Veracruz | Banderilla | 11.9 (8.7, 16.2) | 131.6 | 11.9 | 39.3 | 0 | 23.6 | 0.4 | 1253.6 | 53 | No | No |
| Yucatán | Panabá | 11.8 (8.3, 16.8) | 148.3 | 10.7 | 44.4 | 0 | 21.7 | 26.1 | 11.7 | 45.8 | No | No |
| Yucatán | Tekit | 11.8 (8.4, 16.6) | 43.7 | 10.7 | 44.4 | 0 | 21.7 | 52.1 | 36.4 | 53 | No | No |
| Yucatán | Chumayel | 11.8 (8.5, 16.2) | 90.7 | 10.7 | 44.4 | 0 | 21.7 | 89.9 | 39.5 | 59.9 | No | No |
| Yucatán | Chocholá | 11.6 (8.2, 16.5) | 29.4 | 10.7 | 44.4 | 0 | 21.7 | 31.6 | 16 | 48 | No | No |
| Puebla | Tlaltenango | 11.6 (8.8, 15.3) | 231.6 | 9 | 32.7 | 0 | 15.4 | 0.5 | 331.7 | 42.1 | No | No |
| Oaxaca | San Pedro Mártir | 11.6 (7.9, 17) | 173.6 | 10.5 | 30.4 | 0 | 17.1 | 54.2 | 223.5 | 31.7 | No | Yes |
| Guanajuato | Cuerámaro | 11.6 (8.7, 15.4) | 173.1 | 9.9 | 29.9 | 5.4 | 18.4 | 0.1 | 108.8 | 42.4 | Yes | No |
| Tlaxcala | Tocatlán | 11.5 (8.8, 15.1) | 130.5 | 9.3 | 32.1 | 0 | 13 | 0.3 | 410 | 46 | No | No |
| Yucatán | Xocchel | 11.5 (8.4, 15.6) | 125.8 | 10.7 | 44.4 | 0 | 21.7 | 53.1 | 31.4 | 46.1 | No | No |
| Yucatán | Buctzotz | 11.3 (8, 15.9) | 130.7 | 10.7 | 44.4 | 0 | 21.7 | 28.7 | 13.6 | 48.5 | No | No |
| Tlaxcala | Benito Juárez | 11.2 (8.3, 15.1) | 156.9 | 9.3 | 32.1 | 26.2 | 13 | 0.1 | 235.8 | 46.9 | No | No |
| Michoacán | Huandacareo | 11.2 (7.9, 15.8) | 193.7 | 9.9 | 31.6 | 12.1 | 19.2 | 0.3 | 121.8 | 42 | Yes | No |
| Yucatán | Dzilam de Bravo | 11.1 (7.6, 16) | 158.7 | 10.7 | 44.4 | 0 | 21.7 | 4.7 | 6.3 | 37.6 | No | No |
| Veracruz | Cosautlán de Carvajal | 11 (8.1, 15) | 176.3 | 11.9 | 39.3 | 0 | 23.6 | 0.1 | 213.5 | 43.9 | No | No |
| Veracruz | Jilotepec | 10.9 (8.1, 14.6) | 169.6 | 11.9 | 39.3 | 8.9 | 23.6 | 0.3 | 296.4 | 47.9 | No | No |
| Oaxaca | Guelatao de Juárez | 10.8 (8.1, 14.4) | 251.3 | 10.5 | 30.4 | 0 | 17.1 | 15.4 | 117.7 | 52.5 | No | No |
| Yucatán | Santa Elena | 10.7 (7.3, 15.6) | 153.2 | 10.7 | 44.4 | 38.3 | 21.7 | 83.4 | 7.9 | 43.7 | No | No |
| Zacatecas | Juchipila | 10.6 (7.3, 15.4) | 238 | 10.9 | 33.5 | 11.3 | 18.9 | 0.2 | 37.3 | 39.5 | Yes | No |
| Veracruz | Naranjal | 10.6 (7.7, 14.6) | 240.5 | 11.9 | 39.3 | 0 | 23.6 | 6.5 | 245.2 | 43.4 | No | No |
| Yucatán | Mayapán | 10.5 (7.7, 14.4) | 98.8 | 10.7 | 44.4 | 0 | 21.7 | 98.3 | 39.5 | 45 | No | No |
| Oaxaca | Santa María la Asunción | 10.4 (7.4, 14.8) | 148.1 | 10.5 | 30.4 | 49.4 | 17.1 | 97.4 | 426.5 | 36.6 | No | No |
| Veracruz | Huiloapan de Cuauhtémoc | 10.4 (7.6, 14.2) | 99.8 | 11.9 | 39.3 | 20 | 23.6 | 3 | 385.7 | 40.5 | No | No |
| Zacatecas | Juan Aldama | 10.4 (7.2, 14.9) | 165 | 10.9 | 33.5 | 7.2 | 18.9 | 0.3 | 35 | 42.2 | Yes | No |
| Veracruz | Las Vigas de Ramírez | 10.4 (7.7, 13.9) | 75 | 11.9 | 39.3 | 0 | 23.6 | 0.1 | 197.7 | 42.1 | No | No |
| Michoacán | Pajacuarán | 10.3 (7.7, 13.9) | 211.8 | 9.9 | 31.6 | 24.4 | 19.2 | 0.2 | 114.1 | 44.6 | Yes | No |
| Yucatán | Dzitás | 10.3 (7, 15.2) | 358.7 | 10.7 | 44.4 | 39.9 | 21.7 | 53.4 | 12.2 | 43.9 | Yes | No |
| Michoacán | Salvador Escalante | 10.3 (7.5, 14.1) | 150.4 | 9.9 | 31.6 | 33.4 | 19.2 | 0.2 | 101.6 | 40.9 | Yes | No |
| Yucatán | Ucú | 10.2 (7.4, 14.1) | 117.1 | 10.7 | 44.4 | 0 | 21.7 | 25.7 | 28.1 | 53.7 | No | No |
| Puebla | Caxhuacan | 10.2 (7.4, 14.1) | 121.5 | 9 | 32.7 | 40.5 | 15.4 | 87 | 260 | 38.1 | No | No |
| Jalisco | Ahualulco de Mercado | 10.1 (7.3, 14) | 185.5 | 7.6 | 35.2 | 19.2 | 14 | 0.1 | 85.3 | 53 | No | No |
| Yucatán | Uayma | 10.1 (7.3, 14.1) | 78.4 | 10.7 | 44.4 | 39.2 | 21.7 | 91.5 | 21.6 | 43.3 | No | No |
| Yucatán | Sudzal | 10.1 (7.1, 14.2) | 87.5 | 10.7 | 44.4 | 0 | 21.7 | 59.3 | 7.7 | 43.1 | No | No |
| Puebla | Pahuatlán | 10.1 (7.2, 14.2) | 130 | 9 | 32.7 | 30.6 | 15.4 | 51.8 | 223.5 | 45.1 | Yes | No |
| Yucatán | Kantunil | 10 (7.3, 13.9) | 111.1 | 10.7 | 44.4 | 0 | 21.7 | 42.8 | 27.3 | 36.9 | No | No |
| Sonora | Huásabas | 10 (6.7, 15) | 148.4 | 11.3 | 44 | 0 | 24.6 | 0 | 1.1 | 44.2 | No | Yes |
| Oaxaca | San Andrés Zabache | 10 (7, 14.2) | 418.4 | 10.5 | 30.4 | 209.2 | 17.1 | 13.2 | 104.7 | 23.2 | No | Yes |
| Oaxaca | San Pedro Huilotepec | 10 (7.4, 13.5) | 142.7 | 10.5 | 30.4 | 0 | 17.1 | 54.1 | 118.7 | 46.1 | No | No |
| Puebla | Ixtepec | 9.9 (7.2, 13.8) | 92.5 | 9 | 32.7 | 0 | 15.4 | 95.9 | 355 | 38.3 | Yes | No |
| Michoacán | Purépero | 9.9 (7.5, 13.2) | 204.1 | 9.9 | 31.6 | 9.3 | 19.2 | 0.4 | 79.2 | 46.9 | Yes | No |
| Puebla | Ayotoxco de Guerrero | 9.9 (7.5, 13.2) | 200 | 9 | 32.7 | 0 | 15.4 | 27.2 | 80.2 | 43.5 | Yes | No |
| Yucatán | Cuzamá | 9.9 (7.1, 13.8) | 56.4 | 10.7 | 44.4 | 0 | 21.7 | 59.9 | 55.4 | 50.3 | No | No |
| Oaxaca | San Blas Atempa | 9.9 (7.2, 13.6) | 508.4 | 10.5 | 30.4 | 8.3 | 17.1 | 87.7 | 88.4 | 50.1 | No | No |
| Oaxaca | Reforma de Pineda | 9.9 (6.9, 14.1) | 146.6 | 10.5 | 30.4 | 97.8 | 17.1 | 3.4 | 81.6 | 42.4 | No | Yes |
| Oaxaca | San Pedro Comitancillo | 9.9 (6.9, 14.1) | 162.2 | 10.5 | 30.4 | 97.3 | 17.1 | 49.4 | 90.5 | 44.1 | No | No |
| Tlaxcala | Tetlatlahuca | 9.7 (7.2, 13.2) | 134.9 | 9.3 | 32.1 | 10.4 | 13 | 0.2 | 513.2 | 48.1 | Yes | No |
| Oaxaca | San Pedro Ixcatlán | 9.7 (7.1, 13.1) | 147.7 | 10.5 | 30.4 | 0 | 17.1 | 89.6 | 114.4 | 35.6 | No | No |
| Yucatán | Mocochá | 9.6 (7, 13.2) | 83.4 | 10.7 | 44.4 | 0 | 21.7 | 13.6 | 68.1 | 52.8 | No | No |
| Yucatán | Sucilá | 9.6 (6.7, 13.8) | 252.3 | 10.7 | 44.4 | 0 | 21.7 | 47.7 | 12.8 | 49.4 | No | No |
| Sonora | Trincheras | 9.6 (6.4, 14.3) | 169.1 | 11.3 | 44 | 169.1 | 24.6 | 0.1 | 0.5 | 48.1 | No | No |
| Guanajuato | Tarandacuao | 9.6 (7.1, 13) | 191.5 | 9.9 | 29.9 | 23.9 | 18.4 | 0.1 | 101.5 | 43.3 | Yes | No |
| Yucatán | Cenotillo | 9.5 (6.4, 14) | 141.6 | 10.7 | 44.4 | 70.8 | 21.7 | 37.3 | 7.2 | 42.4 | No | No |
| Sonora | Oquitoa | 9.4 (6.3, 14.2) | 1278 | 11.3 | 44 | 319.5 | 24.6 | 0.7 | 0.4 | 42.2 | No | No |
| Yucatán | Ixil | 9.4 (6.7, 13.2) | 0 | 10.7 | 44.4 | 0 | 21.7 | 15.4 | 29.8 | 56.2 | No | No |
| Yucatán | San Felipe | 9.4 (6.6, 13.4) | 71.9 | 10.7 | 44.4 | 0 | 21.7 | 8.4 | 4.3 | 49.7 | No | No |
| Veracruz | Rafael Lucio | 9.4 (7, 12.7) | 118.3 | 11.9 | 39.3 | 19.7 | 23.6 | 0.2 | 700.3 | 52.2 | No | No |
| Yucatán | Temax | 9.4 (6.7, 13.2) | 178.6 | 10.7 | 44.4 | 39.7 | 21.7 | 29 | 21.4 | 43.9 | No | No |
| Michoacán | Nahuatzen | 9.4 (7, 12.6) | 113.3 | 9.9 | 31.6 | 5.7 | 19.2 | 40.9 | 92.3 | 37.6 | Yes | No |
| Veracruz | Rafael Delgado | 9.4 (7, 12.5) | 143.3 | 11.9 | 39.3 | 7.2 | 23.6 | 49.8 | 874.1 | 47.8 | No | No |
| Puebla | Tepeyahualco de Cuauhtémoc | 9.3 (6.9, 12.6) | 84 | 9 | 32.7 | 0 | 15.4 | 0.7 | 226 | 48.1 | No | No |
| Oaxaca | Santa María Xadani | 9.3 (6.8, 12.6) | 159.4 | 10.5 | 30.4 | 19.9 | 17.1 | 96.9 | 102.2 | 52.3 | No | No |
| Oaxaca | San Lorenzo Cuaunecuiltitla | 9.3 (6.7, 12.7) | 211.9 | 10.5 | 30.4 | 211.9 | 17.1 | 93.5 | 88 | 32.4 | No | No |
| Yucatán | Dzilam González | 9.2 (6.4, 13.3) | 180.1 | 10.7 | 44.4 | 22.5 | 21.7 | 17.8 | 12.1 | 44.6 | No | No |
| Guerrero | Cuajinicuilapa | 9.2 (6.7, 12.8) | 112.4 | 11.1 | 34.1 | 0 | 17.9 | 5.7 | 43.2 | 45.4 | Yes | Yes |
| Sonora | Benjamín Hill | 9.2 (6.3, 13.6) | 136.4 | 11.3 | 44 | 27.3 | 24.6 | 0.3 | 3.7 | 40.9 | No | No |
| Veracruz | Tlilapan | 9.2 (6.7, 12.7) | 278.9 | 11.9 | 39.3 | 0 | 23.6 | 38 | 469 | 46.4 | No | No |
| Tlaxcala | Cuaxomulco | 9.2 (6.7, 12.6) | 112.6 | 9.3 | 32.1 | 28.2 | 13 | 1.1 | 316.3 | 46.4 | No | No |
| Yucatán | Sotuta | 9.2 (6.5, 13) | 53 | 10.7 | 44.4 | 0 | 21.7 | 56.3 | 16.3 | 46.4 | No | No |
| SLP | Tanquián de Escobedo | 9.2 (6.6, 12.6) | 89.4 | 10.8 | 33 | 0 | 19.2 | 17.3 | 105.4 | 43.3 | No | No |
| Guanajuato | Apaseo el Alto | 9.2 (6.9, 12.2) | 121.5 | 9.9 | 29.9 | 9.2 | 18.4 | 0.2 | 183.1 | 45.7 | Yes | No |
| Sonora | Cumpas | 9.1 (6.1, 13.7) | 86.9 | 11.3 | 44 | 43.4 | 24.6 | 0 | 3 | 41.7 | No | No |
| Oaxaca | San Nicolás Hidalgo | 9 (6.4, 12.8) | 163.4 | 10.5 | 30.4 | 0 | 17.1 | 0.1 | 85.9 | 24.2 | No | Yes |
| Veracruz | Coetzala | 9 (6.6, 12.3) | 139.2 | 11.9 | 39.3 | 0 | 23.6 | 35.4 | 236.9 | 40.4 | No | No |
| Oaxaca | Santa Cruz Papalutla | 8.9 (6.6, 12.1) | 201.6 | 10.5 | 30.4 | 0 | 17.1 | 35.6 | 144 | 50.2 | No | Yes |
| Oaxaca | San Jerónimo Tecóatl | 8.9 (6.6, 12.1) | 195.3 | 10.5 | 30.4 | 0 | 17.1 | 78.8 | 89.2 | 41.3 | No | No |
| México | Chiautla | 8.9 (6.6, 11.9) | 263.1 | 9 | 32.7 | 0 | 15.5 | 0.8 | 1453.6 | 50.1 | No | No |
| Michoacán | Cherán | 8.9 (6.7, 11.7) | 144.2 | 9.9 | 31.6 | 8.5 | 19.2 | 26.8 | 85.8 | 47.9 | Yes | No |
| Michoacán | Quiroga | 8.8 (6.7, 11.7) | 120.4 | 9.9 | 31.6 | 5.5 | 19.2 | 31.9 | 130.6 | 53.1 | Yes | No |
| Veracruz | Espinal | 8.8 (6.6, 11.7) | 175.9 | 11.9 | 39.3 | 5.7 | 23.6 | 39 | 113.3 | 38.9 | Yes | No |
| Oaxaca | San Miguel Ejutla | 8.8 (6.5, 12) | 155 | 10.5 | 30.4 | 0 | 17.1 | 5.2 | 94.3 | 41.7 | No | Yes |
| Veracruz | Cuichapa | 8.8 (6.5, 11.9) | 85.7 | 11.9 | 39.3 | 12.2 | 23.6 | 1.7 | 352.6 | 42.1 | No | No |
| Jalisco | Arandas | 8.8 (6.5, 11.9) | 91.2 | 7.6 | 35.2 | 8.1 | 14 | 0.4 | 81.2 | 52.4 | Yes | No |
| Sonora | Ures | 8.7 (5.8, 13.2) | 146.4 | 11.3 | 44 | 29.3 | 24.6 | 0.1 | 2.8 | 47.7 | Yes | No |
| Puebla | Atlequizayan | 8.7 (6, 12.6) | 115.1 | 9 | 32.7 | 57.6 | 15.4 | 98.2 | 206.1 | 32 | No | No |
| Guanajuato | Santiago Maravatío | 8.7 (6.2, 12.2) | 240.5 | 9.9 | 29.9 | 0 | 18.4 | 0.1 | 81.6 | 28.6 | No | No |
| Oaxaca | Santa Cruz Acatepec | 8.7 (6.2, 12.3) | 0 | 10.5 | 30.4 | 0 | 17.1 | 91.9 | 218 | 40.7 | No | No |
| Yucatán | Río Lagartos | 8.7 (6, 12.5) | 39.5 | 10.7 | 44.4 | 0 | 21.7 | 12.7 | 10.4 | 44 | No | No |
| Michoacán | Los Reyes | 8.6 (6.5, 11.4) | 138 | 9.9 | 31.6 | 9 | 19.2 | 19.4 | 145 | 53.3 | Yes | No |
| Yucatán | Opichén | 8.6 (6.2, 12) | 43.9 | 10.7 | 44.4 | 0 | 21.7 | 76.6 | 26.2 | 48.7 | No | No |
| Sonora | Alamos | 8.6 (5.8, 12.7) | 104.9 | 11.3 | 44 | 16.6 | 24.6 | 5.4 | 4 | 39.8 | Yes | No |
| Oaxaca | San Jerónimo Tlacochahuaya | 8.6 (6.3, 11.8) | 202.7 | 10.5 | 30.4 | 25.3 | 17.1 | 46.9 | 149.8 | 53.5 | No | No |
| Jalisco | Tizapán el Alto | 8.6 (6.3, 11.7) | 143.7 | 7.6 | 35.2 | 14.4 | 14 | 0.2 | 108.1 | 46.4 | Yes | No |
| Puebla | Yaonáhuac | 8.6 (6.4, 11.4) | 253.9 | 9 | 32.7 | 0 | 15.4 | 46.7 | 268.4 | 46.3 | No | No |
| Puebla | Tepango de Rodríguez | 8.6 (6.4, 11.5) | 193.1 | 9 | 32.7 | 0 | 15.4 | 85 | 137.4 | 36.1 | No | Yes |
| Michoacán | Tangancícuaro | 8.5 (6.3, 11.7) | 218.5 | 9.9 | 31.6 | 22.3 | 19.2 | 5.5 | 87.3 | 49 | Yes | No |
| Michoacán | Lagunillas | 8.5 (6.4, 11.4) | 111.4 | 9.9 | 31.6 | 0 | 19.2 | 0.1 | 76.7 | 41.5 | Yes | No |
| Sonora | Banámichi | 8.5 (5.7, 12.8) | 83.5 | 11.3 | 44 | 0 | 24.6 | 0.1 | 2 | 44 | No | Yes |
| Veracruz | Acatlán | 8.5 (6.2, 11.7) | 245.3 | 11.9 | 39.3 | 0 | 23.6 | 0.2 | 172.3 | 41.4 | No | No |
| Tlaxcala | Tzompantepec | 8.5 (6.1, 11.7) | 63.1 | 9.3 | 32.1 | 36.1 | 13 | 0.8 | 426 | 50 | Yes | No |
| Oaxaca | Tlalixtac de Cabrera | 8.4 (6.3, 11.3) | 99.1 | 10.5 | 30.4 | 14.2 | 17.1 | 12.6 | 125.2 | 54 | No | No |
| Veracruz | Chocamán | 8.4 (6.1, 11.4) | 106.1 | 11.9 | 39.3 | 0 | 23.6 | 0.5 | 476.1 | 48.5 | No | No |
| Oaxaca | Taniche | 8.2 (5.7, 11.9) | 0 | 10.5 | 30.4 | 0 | 17.1 | 0.3 | 76.7 | 38.2 | No | Yes |
| Sonora | Nacozari de García | 8.2 (5.5, 12.3) | 34.1 | 11.3 | 44 | 0 | 24.6 | 1.1 | 8 | 47.5 | Yes | No |
| Puebla | Tianguismanalco | 8.2 (6, 11.2) | 190.5 | 9 | 32.7 | 12.7 | 15.4 | 9.2 | 95.9 | 50.5 | Yes | No |
| Morelos | Tetecala | 8.2 (5.7, 11.9) | 111.2 | 12 | 33.5 | 37.1 | 17.5 | 0.3 | 114.8 | 49 | Yes | No |
| Sonora | Bacadéhuachi | 8.2 (5.4, 12.4) | 0 | 11.3 | 44 | 0 | 24.6 | 0 | 1 | 28.6 | No | Yes |
| México | Tonatico | 8.2 (5.9, 11.2) | 191.7 | 9 | 32.7 | 11.3 | 15.5 | 0.2 | 136.7 | 48.2 | No | No |
| Oaxaca | San Pedro Apóstol | 8.2 (5.7, 11.6) | 89.8 | 10.5 | 30.4 | 0 | 17.1 | 2.5 | 169.5 | 39.8 | No | Yes |
| Veracruz | Coahuitlán | 8.2 (6, 11.1) | 121.3 | 11.9 | 39.3 | 0 | 23.6 | 53.5 | 205.1 | 30.5 | No | No |
| Zacatecas | Tabasco | 8.1 (5.9, 11.2) | 149.5 | 10.9 | 33.5 | 10 | 18.9 | 0.3 | 37.7 | 40.4 | Yes | No |
| Yucatán | Teabo | 8.1 (5.9, 11.2) | 96 | 10.7 | 44.4 | 48 | 21.7 | 81.4 | 29.2 | 61.7 | No | No |
| Guerrero | Petatlán | 8.1 (5.8, 11.3) | 103.9 | 11.1 | 34.1 | 17.9 | 17.9 | 0.5 | 22.4 | 46.5 | Yes | No |
| Michoacán | Tangamandapio | 8 (6, 10.7) | 117.9 | 9.9 | 31.6 | 28.1 | 19.2 | 34.5 | 92.6 | 44.3 | Yes | No |
| Sonora | Granados | 8 (5.2, 12.3) | 0 | 11.3 | 44 | 248.1 | 24.6 | 0 | 2.9 | 45.8 | No | Yes |
| Guerrero | Benito Juárez | 8 (5.6, 11.4) | 173.1 | 11.1 | 34.1 | 9.1 | 17.9 | 0.6 | 66.6 | 46.7 | No | No |
| Yucatán | Chacsinkín | 8 (5.7, 11.1) | 55.3 | 10.7 | 44.4 | 0 | 21.7 | 96.3 | 26.3 | 45.3 | No | No |
| Oaxaca | Rojas de Cuauhtémoc | 8 (5.6, 11.3) | 117.8 | 10.5 | 30.4 | 0 | 17.1 | 1.1 | 87.9 | 48.3 | No | No |
| Oaxaca | San Juan Guelavía | 8 (5.6, 11.3) | 124.6 | 10.5 | 30.4 | 0 | 17.1 | 69.9 | 101.1 | 39 | No | No |
| Sonora | Mazatán | 7.9 (5.3, 11.9) | 206.8 | 11.3 | 44 | 103.4 | 24.6 | 0.1 | 1.8 | 52.3 | No | No |
| Oaxaca | Santo Domingo Tomaltepec | 7.9 (5.8, 10.8) | 93.4 | 10.5 | 30.4 | 46.7 | 17.1 | 21.9 | 97.8 | 50 | No | No |
| Sonora | Huachinera | 7.8 (5.3, 11.5) | 328.2 | 11.3 | 44 | 0 | 24.6 | 0.1 | 1 | 43 | No | Yes |
| Sonora | Arivechi | 7.8 (5.3, 11.5) | 221.2 | 11.3 | 44 | 0 | 24.6 | 0.4 | 1.6 | 32.5 | No | Yes |
| Yucatán | Tunkás | 7.8 (5.3, 11.5) | 122.9 | 10.7 | 44.4 | 0 | 21.7 | 52.1 | 8.5 | 41.6 | No | No |
| Yucatán | Abalá | 7.8 (5.4, 11.2) | 224.1 | 10.7 | 44.4 | 0 | 21.7 | 65.2 | 22.2 | 49.5 | No | No |
| Oaxaca | Magdalena Teitipac | 7.8 (5.8, 10.4) | 110.1 | 10.5 | 30.4 | 0 | 17.1 | 93.2 | 116 | 44.7 | No | No |
| Sonora | Cucurpe | 7.8 (5.1, 11.9) | 0 | 11.3 | 44 | 0 | 24.6 | 0.2 | 0.6 | 52.3 | No | No |
| Sonora | Santa Cruz | 7.8 (5.4, 11.2) | 243.3 | 11.3 | 44 | 0 | 24.6 | 0.6 | 1.8 | 44.3 | No | No |
| SLP | El Naranjo | 7.8 (5.5, 10.9) | 186.9 | 10.8 | 33 | 13.8 | 19.2 | 0.7 | 26.7 | 42.7 | Yes | No |
| Puebla | Aljojuca | 7.7 (5.7, 10.5) | 133.4 | 9 | 32.7 | 0 | 15.4 | 0.2 | 124.7 | 37.3 | No | No |
| Sonora | Divisaderos | 7.7 (5.3, 11.4) | 178.3 | 11.3 | 44 | 0 | 24.6 | 0.1 | 1.9 | 36.3 | No | Yes |
| Sonora | Bacoachi | 7.7 (5.3, 11.4) | 166 | 11.3 | 44 | 0 | 24.6 | 0 | 1.3 | 42 | No | No |
| Sonora | Villa Hidalgo | 7.7 (5.3, 11.4) | 167.8 | 11.3 | 44 | 0 | 24.6 | 0.1 | 1 | 36.7 | No | No |
| Sonora | Altar | 7.7 (5.3, 11.2) | 81.8 | 11.3 | 44 | 0 | 24.6 | 0.6 | 2.1 | 46.8 | No | No |
| Jalisco | Tuxpan | 7.7 (5.6, 10.6) | 123.2 | 7.6 | 35.2 | 0 | 14 | 0.6 | 47.6 | 48.7 | Yes | No |
| Oaxaca | San Dionisio Ocotlán | 7.6 (5.6, 10.5) | 122.2 | 10.5 | 30.4 | 0 | 17.1 | 3 | 106.8 | 39.9 | No | Yes |
| Oaxaca | San Martín de los Cansecos | 7.6 (5.5, 10.6) | 0 | 10.5 | 30.4 | 0 | 17.1 | 0.4 | 102.4 | 37.7 | No | Yes |
| Puebla | Coxcatlán | 7.6 (5.6, 10.3) | 150.6 | 9 | 32.7 | 15.1 | 15.4 | 35.9 | 82.1 | 40.9 | Yes | No |
| Yucatán | Cuncunul | 7.6 (5.4, 10.7) | 0 | 10.7 | 44.4 | 0 | 21.7 | 79.8 | 11.6 | 43.4 | No | No |
| Sonora | Suaqui Grande | 7.6 (5.1, 11.4) | 120.5 | 11.3 | 44 | 120.5 | 24.6 | 0 | 1.2 | 45.6 | No | No |
| Oaxaca | San Sebastián Abasolo | 7.6 (5.3, 10.8) | 71.4 | 10.5 | 30.4 | 0 | 17.1 | 32.4 | 125.8 | 45.3 | No | No |
| Tlaxcala | Lázaro Cárdenas | 7.6 (5.7, 10) | 208.4 | 9.3 | 32.1 | 0 | 13 | 0.2 | 122 | 46.1 | No | No |
| Guerrero | Florencio Villarreal | 7.5 (5.5, 10.3) | 155.6 | 11.1 | 34.1 | 16.4 | 17.9 | 0.9 | 73.3 | 43.5 | No | No |
| Oaxaca | San Ildefonso Villa Alta | 7.5 (5.3, 10.6) | 210.6 | 10.5 | 30.4 | 84.2 | 17.1 | 68.4 | 35.3 | 44 | Yes | Yes |
| Sonora | Rayón | 7.5 (5, 11.1) | 83.2 | 11.3 | 44 | 0 | 24.6 | 0 | 1.6 | 47.9 | No | No |
| Veracruz | Otatitlán | 7.5 (5.5, 10.2) | 200 | 11.9 | 39.3 | 0 | 23.6 | 1.7 | 114.4 | 43.9 | No | No |
| Veracruz | Miahuatlán | 7.5 (5.5, 10.1) | 561.6 | 11.9 | 39.3 | 0 | 23.6 | 0.2 | 159 | 42.2 | No | No |
| Jalisco | Jamay | 7.5 (5.5, 10.2) | 124.2 | 7.6 | 35.2 | 0 | 14 | 0.3 | 152.1 | 48.2 | No | No |
| Tamaulipas | Miguel Alemán | 7.5 (5, 11.1) | 68.7 | 12.8 | 41 | 5.3 | 18.4 | 0.4 | 43 | 48.9 | Yes | No |
| Michoacán | Indaparapeo | 7.5 (5.4, 10.3) | 57 | 9.9 | 31.6 | 19 | 19.2 | 0.2 | 96.3 | 43.7 | Yes | No |
| Sonora | Atil | 7.5 (4.9, 11.4) | 0 | 11.3 | 44 | 0 | 24.6 | 0.7 | 1.9 | 37.7 | No | Yes |
| Guerrero | Teloloapan | 7.4 (5.4, 10.2) | 164.9 | 11.1 | 34.1 | 8.4 | 17.9 | 1.8 | 56.8 | 36.3 | Yes | No |
| Oaxaca | Matías Romero Avendaño | 7.4 (5.2, 10.4) | 188.3 | 10.5 | 30.4 | 38.4 | 17.1 | 13.5 | 29.4 | 0 | Yes | No |
| Michoacán | Numarán | 7.4 (5.5, 9.9) | 120.4 | 9.9 | 31.6 | 0 | 19.2 | 0.1 | 127.3 | 40.2 | No | No |
| Coahuila | Sabinas | 7.4 (5.3, 10.3) | 134.4 | 12.4 | 38.6 | 9.1 | 22.4 | 0.2 | 32.1 | 50 | Yes | No |
| SLP | Huehuetlán | 7.4 (5.2, 10.3) | 168.8 | 10.8 | 33 | 39.7 | 19.2 | 67.7 | 221.5 | 34.5 | No | No |
| Michoacán | Tingambato | 7.4 (5.6, 9.7) | 176.9 | 9.9 | 31.6 | 0 | 19.2 | 6.9 | 79.1 | 45.4 | No | No |
| Oaxaca | San Pedro Ocopetatillo | 7.3 (5, 10.7) | 0 | 10.5 | 30.4 | 191.2 | 17.1 | 94.4 | 119.7 | 40.7 | No | No |
| Oaxaca | Santa Cruz Tacache de Mina | 7.3 (5.3, 10.1) | 59 | 10.5 | 30.4 | 0 | 17.1 | 0.2 | 97.1 | 28.5 | No | Yes |
| Yucatán | Tixmehuac | 7.3 (5.1, 10.5) | 298.3 | 10.7 | 44.4 | 0 | 21.7 | 91.5 | 20.9 | 43.1 | No | No |
| Sonora | Bacerac | 7.3 (5, 10.6) | 99.7 | 11.3 | 44 | 0 | 24.6 | 0.2 | 1 | 34.2 | No | No |
| Yucatán | Tahdziú | 7.3 (5.4, 9.9) | 78.9 | 10.7 | 44.4 | 0 | 21.7 | 99.1 | 21.2 | 54 | No | No |
| Morelos | Amacuzac | 7.2 (5.2, 10.1) | 168.9 | 12 | 33.5 | 16.9 | 17.5 | 0.3 | 151.6 | 43.6 | No | No |
| Oaxaca | Teotitlán de Flores Magón | 7.2 (5.4, 9.6) | 173.2 | 10.5 | 30.4 | 15.7 | 17.1 | 19.8 | 68.6 | 47.4 | Yes | No |
| Michoacán | Chilchota | 7.2 (5.4, 9.6) | 130.9 | 9.9 | 31.6 | 4.1 | 19.2 | 58.4 | 128.1 | 48.8 | No | No |
| Jalisco | Casimiro Castillo | 7.2 (5.3, 9.8) | 183.3 | 7.6 | 35.2 | 13.6 | 14 | 0.2 | 41.3 | 45.8 | Yes | No |
| Oaxaca | Magdalena Ocotlán | 7.2 (5.2, 9.9) | 0 | 10.5 | 30.4 | 0 | 17.1 | 15.4 | 110.9 | 43.6 | No | Yes |
| Oaxaca | San Miguel el Grande | 7.2 (5.3, 9.8) | 142.1 | 10.5 | 30.4 | 0 | 17.1 | 59.9 | 39.8 | 35.1 | No | No |
| Oaxaca | Villa Talea de Castro | 7.2 (5.1, 10.1) | 318.3 | 10.5 | 30.4 | 0 | 17.1 | 52.7 | 37.3 | 54 | No | Yes |
| Oaxaca | San Cristóbal Lachirioag | 7.2 (5.1, 10.1) | 515.5 | 10.5 | 30.4 | 0 | 17.1 | 93.8 | 71.6 | 43.5 | No | Yes |
| Puebla | Zapotitlán de Méndez | 7.2 (5, 10.3) | 0 | 9 | 32.7 | 0 | 15.4 | 75.4 | 268.7 | 39.3 | Yes | Yes |
| Oaxaca | Santo Tomás Mazaltepec | 7.2 (5.3, 9.6) | 232.8 | 10.5 | 30.4 | 0 | 17.1 | 53.7 | 59.8 | 57.5 | No | No |
| Oaxaca | San Mateo Etlatongo | 7.1 (5.2, 9.8) | 260.4 | 10.5 | 30.4 | 130.2 | 17.1 | 1.4 | 47.1 | 34.5 | No | Yes |
| Tamaulipas | Camargo | 7.1 (5, 10) | 122.3 | 12.8 | 41 | 0 | 18.4 | 0.3 | 16.9 | 45.8 | Yes | No |
| Yucatán | Timucuy | 7.1 (5.1, 9.9) | 80.2 | 10.7 | 44.4 | 0 | 21.7 | 88.1 | 54.7 | 54.3 | No | No |
| Zacatecas | Tlaltenango de Sánchez Román | 7 (5, 9.8) | 86.5 | 10.9 | 33.5 | 5.8 | 18.9 | 1.6 | 35.7 | 45.2 | Yes | No |
| Chiapas | Las Rosas | 7 (5.2, 9.5) | 121.4 | 7.8 | 29 | 0 | 16.2 | 5.9 | 120.4 | 47.5 | Yes | No |
| Hidalgo | Atlapexco | 7 (4.8, 10) | 45.1 | 12.9 | 31.2 | 7.5 | 17.9 | 78.2 | 139.5 | 34.4 | Yes | No |
| Oaxaca | San Pedro Tapanatepec | 7 (5.1, 9.6) | 191.4 | 10.5 | 30.4 | 10.1 | 17.1 | 3.3 | 15.2 | 44 | Yes | No |
| Oaxaca | Santa Ana | 6.9 (5.1, 9.3) | 76.2 | 10.5 | 30.4 | 0 | 17.1 | 0.3 | 39.3 | 32.9 | No | No |
| Yucatán | Tixcacalcupul | 6.9 (5.1, 9.4) | 94.7 | 10.7 | 44.4 | 0 | 21.7 | 94.1 | 14.2 | 38.3 | No | No |
| Yucatán | Tekal de Venegas | 6.9 (4.7, 10) | 0 | 10.7 | 44.4 | 54.6 | 21.7 | 56.5 | 13.1 | 39 | No | No |
| Sonora | Huépac | 6.8 (4.6, 10) | 126.3 | 11.3 | 44 | 0 | 24.6 | 0.1 | 2.2 | 39.3 | No | Yes |
| Guerrero | Alpoyeca | 6.8 (4.9, 9.5) | 277.1 | 11.1 | 34.1 | 25.2 | 17.9 | 17.8 | 70.9 | 37.4 | No | No |
| Puebla | Chapulco | 6.8 (4.9, 9.4) | 178.8 | 9 | 32.7 | 22.4 | 15.4 | 1.6 | 89 | 49.4 | No | Yes |
| Oaxaca | Santa Cruz Xitla | 6.8 (4.9, 9.3) | 176.8 | 10.5 | 30.4 | 70.7 | 17.1 | 67.8 | 83.6 | 36.4 | No | No |
| Durango | Nuevo Ideal | 6.8 (4.8, 9.5) | 100.3 | 10.9 | 37.1 | 5.9 | 20.2 | 0.2 | 14.7 | 41.6 | Yes | No |
| Guerrero | Apaxtla | 6.8 (4.8, 9.5) | 185.6 | 11.1 | 34.1 | 14.3 | 17.9 | 0.8 | 17.8 | 35.6 | No | No |
| Jalisco | Atotonilco el Alto | 6.8 (4.9, 9.3) | 110.6 | 7.6 | 35.2 | 10.1 | 14 | 0.2 | 118.4 | 47.1 | Yes | No |
| Jalisco | San Ignacio Cerro Gordo | 6.8 (4.7, 9.8) | 50.5 | 7.6 | 35.2 | 25.3 | 14 | 0.3 | 83.2 | 48.3 | Yes | No |
| Michoacán | Santa Ana Maya | 6.7 (5, 9) | 117.7 | 9.9 | 31.6 | 0 | 19.2 | 0.1 | 119.8 | 35 | No | No |
| Veracruz | Chinameca | 6.7 (5.1, 8.9) | 166.2 | 11.9 | 39.3 | 8.7 | 23.6 | 2 | 93.1 | 45.2 | No | No |
| Oaxaca | San Miguel Suchixtepec | 6.7 (5, 9) | 461.9 | 10.5 | 30.4 | 0 | 17.1 | 67.6 | 38.3 | 39.3 | No | Yes |
| Jalisco | San Martín Hidalgo | 6.7 (4.6, 9.7) | 118.7 | 7.6 | 35.2 | 15.5 | 14 | 0.2 | 81 | 40.2 | No | No |
| Veracruz | Coxquihui | 6.7 (5, 9.1) | 183.4 | 11.9 | 39.3 | 19.3 | 23.6 | 66.7 | 208.7 | 35 | No | No |
| Oaxaca | San Felipe Tejalápam | 6.7 (4.9, 9.1) | 139.2 | 10.5 | 30.4 | 19.9 | 17.1 | 1.7 | 79.1 | 46.8 | No | No |
| Veracruz | Filomeno Mata | 6.7 (4.8, 9.3) | 44.5 | 11.9 | 39.3 | 11.1 | 23.6 | 97.6 | 424.3 | 31.9 | No | No |
| Michoacán | Tanhuato | 6.7 (4.9, 9.1) | 289.1 | 9.9 | 31.6 | 28.9 | 19.2 | 1.1 | 67.3 | 41.6 | Yes | No |
| Puebla | Atzala | 6.7 (5, 9) | 118.2 | 9 | 32.7 | 0 | 15.4 | 0.3 | 116.9 | 41.6 | No | No |
| Oaxaca | San Lorenzo Victoria | 6.7 (4.6, 9.6) | 295.4 | 10.5 | 30.4 | 147.7 | 17.1 | 1.5 | 18.1 | 32.4 | No | Yes |
| Guerrero | San Luis Acatlán | 6.7 (4.9, 9.1) | 146.6 | 11.1 | 34.1 | 17.3 | 17.9 | 54.6 | 39.8 | 32.2 | Yes | No |
| Oaxaca | San Juan del Estado | 6.7 (4.8, 9.2) | 217.9 | 10.5 | 30.4 | 0 | 17.1 | 1.2 | 19.6 | 43.8 | No | No |
| Oaxaca | San Miguel Aloápam | 6.7 (4.8, 9.2) | 498.6 | 10.5 | 30.4 | 0 | 17.1 | 98.1 | 15.3 | 39.1 | No | No |
| Michoacán | Huiramba | 6.6 (4.9, 9) | 244.5 | 9.9 | 31.6 | 0 | 19.2 | 0.3 | 111.7 | 41.5 | Yes | No |
| Sonora | Tepache | 6.6 (4.5, 9.6) | 92.4 | 11.3 | 44 | 0 | 24.6 | 0 | 1.6 | 36.6 | No | Yes |
| Sonora | San Pedro de la Cueva | 6.6 (4.5, 9.6) | 82.6 | 11.3 | 44 | 0 | 24.6 | 0.1 | 0.7 | 43.3 | No | No |
| Sonora | Arizpe | 6.6 (4.5, 9.6) | 96.9 | 11.3 | 44 | 0 | 24.6 | 0 | 0.9 | 36.4 | No | No |
| Oaxaca | San Mateo del Mar | 6.6 (4.9, 8.8) | 80.1 | 10.5 | 30.4 | 0 | 17.1 | 98.3 | 165.3 | 42.2 | No | No |
| Oaxaca | Heroica Ciudad de Ejutla de Crespo | 6.6 (4.8, 9) | 206.1 | 10.5 | 30.4 | 22.1 | 17.1 | 2.5 | 65.9 | 40.4 | No | No |
| Oaxaca | Santo Domingo Petapa | 6.6 (4.8, 9) | 176.1 | 10.5 | 30.4 | 52.8 | 17.1 | 36.7 | 27 | 44.3 | No | No |
| Sonora | Bacanora | 6.5 (4.3, 9.9) | 0 | 11.3 | 44 | 0 | 24.6 | 0.3 | 0.7 | 35.8 | No | Yes |
| Sonora | Onavas | 6.5 (4.3, 9.9) | 0 | 11.3 | 44 | 0 | 24.6 | 0.3 | 0.9 | 48.9 | No | No |
| Oaxaca | Santiago Lalopa | 6.5 (4.8, 9) | 313.5 | 10.5 | 30.4 | 0 | 17.1 | 92.5 | 19.4 | 47.3 | No | Yes |
| Oaxaca | Nuevo Zoquiápam | 6.5 (4.8, 9) | 208.3 | 10.5 | 30.4 | 0 | 17.1 | 44.5 | 16.4 | 39.4 | No | No |
| Sonora | Naco | 6.5 (4.6, 9.3) | 99.9 | 11.3 | 44 | 0 | 24.6 | 1 | 5 | 48.9 | No | No |
| Puebla | Ixcaquixtla | 6.5 (4.7, 9.1) | 70.3 | 9 | 32.7 | 0 | 15.4 | 1 | 82 | 45.7 | Yes | No |
| Oaxaca | Santiago Tillo | 6.5 (4.5, 9.3) | 250 | 10.5 | 30.4 | 0 | 17.1 | 5.4 | 30.6 | 47 | No | Yes |
| Oaxaca | Santo Domingo Ixcatlán | 6.5 (4.5, 9.3) | 201.2 | 10.5 | 30.4 | 0 | 17.1 | 24.7 | 36.4 | 33.2 | No | No |
| Oaxaca | San Juan Bautista Valle Nacional | 6.5 (4.6, 9.2) | 249.8 | 10.5 | 30.4 | 6.9 | 17.1 | 57.7 | 33.2 | 31.1 | Yes | No |
| Zacatecas | Momax | 6.4 (4.5, 9.2) | 170.6 | 10.9 | 33.5 | 0 | 18.9 | 0 | 14.9 | 39.9 | No | No |
| Oaxaca | San Bartolomé Quialana | 6.4 (4.7, 8.9) | 60.5 | 10.5 | 30.4 | 0 | 17.1 | 96.8 | 103.7 | 36.5 | No | No |
| Yucatán | Celestún | 6.4 (4.4, 9.4) | 98.8 | 10.7 | 44.4 | 39.5 | 21.7 | 7.3 | 13 | 51.9 | No | No |
| Yucatán | Calotmul | 6.4 (4.4, 9.2) | 281.3 | 10.7 | 44.4 | 0 | 21.7 | 60.3 | 13.7 | 42.4 | No | No |
| Sonora | San Felipe de Jesús | 6.4 (4.2, 9.8) | 0 | 11.3 | 44 | 327.9 | 24.6 | 0.8 | 2.7 | 40.1 | No | Yes |
| Oaxaca | Santa Ana del Valle | 6.4 (4.6, 9) | 401.9 | 10.5 | 30.4 | 0 | 17.1 | 82.6 | 74.1 | 37.1 | No | No |
| Sonora | Pitiquito | 6.4 (4.3, 9.5) | 45.3 | 11.3 | 44 | 15.1 | 24.6 | 3.1 | 1 | 45.9 | No | No |
| Oaxaca | Santa María Guelacé | 6.4 (4.5, 9) | 0 | 10.5 | 30.4 | 348.4 | 17.1 | 21 | 110.2 | 54.5 | No | Yes |

Characteristics are displayed in quintiles of the distribution within 2457 municipalities of the country: Red cell: Quintile 5, no hospital, or hope municipality, Orange cell: Quintile 4, Yellow cell: Quintile 3, Olive cell: Quintile 2, Green cell: Quintile 1, Hospital in the municipality, or not a Hope Municipality.

2020 Data: Hospitals.

2018 Data: State diabetes prevalence, Diabetes Crude Mortality Rate, State hypertension prevalence, Hypertension Crude Mortality Rate, State Obesity Prevalence.

2015 Data: Economically active population, Population density.

2010 Data: Indigenous population.

Supplementary Table 2. Individual factor risk of dying COVID-19 in confirmed symptomatic cases in Mexico

|  | Category | IRR (95% CI) | p value |
| --- | --- | --- | --- |
| Individual Characteristics | <40 y old | 1 |  |
|  | 40-59 y old | 4.37 (4.12, 4.63) | <0.001 |
|  | ≥60 y old | 10.5 (9.96, 11.2) | <0.001 |
|  | Males | 1.52 (1.48, 1.57) | <0.001 |
|  | Obesity | 1.24 (1.20, 1.29) | <0.001 |
|  | Smoker | 0.96 (0.91, 1.01) | 0.133 |
|  | Indigenism | 1.39 (1.25, 1.54) | <0.001 |
| Comorbidities | Chronic kidney disease | 1.45 (1.38, 1.54) | <0.001 |
|  | Diabetes | 1.42 (1.37, 1.46) | <0.001 |
|  | COPD | 1.21 (1.14, 1.30) | <0.001 |
|  | Hypertension | 1.22 (1.18, 1.26) | <0.001 |
|  | Other comorbidities*^¥^ | 1.16 (1.09, 1.24) | <0.001 |
|  | Immunosuppression | 1.23 (1.12, 1.35) | <0.001 |
|  | Cardiovascular diseases | 1.02 (0.96, 1.08) | 0.450 |
|  | Asthma | 0.82 (0.74, 0.90) | <0.001 |
| Institution | Private | 1 |  |
|  | SSA (Public)^1^ | 1.65 (1.45, 1.86) | <0.001 |
|  | IMSS (Public)^2^ | 3.18 (2.81, 3.60) | <0.001 |
|  | ISSSTE (Public)^3^ | 2.49 (2.18, 2.85) | <0.001 |
|  | Other | 1.82 (1.59, 2.09) | <0.001 |
| Days that passed between the onset of symptoms and medical attention | 0-5 days | 1 |  |
|  | 6-14 days | 1.05 (1.02, 1.08) | 0.001 |
|  | ≥15 days | 0.76 (0.63, 0.92) | 0.001 |

^£^Open data from the Ministry of Health. The sample includes 19 845 deaths from the 167 851 accumulated confirmed cases with complete information of June 20, 2020. ^¥^ Other comorbidities*.

^1^SSA: Ministry of Health Hospitals.^2^IMSS: Mexican Social Security Institute. ^3^ISSSTE: The Mexican Civil Service Social Security and Services Institute.

References categories were: <40 years old, females, and not having the condition.

| Supplementary Table 3. Municipal factors associated with Cummulative Incidence and Case Fatality Rates of COVID-19 in symptomatic cases in four moments of the pandemic in Mexico. | | | | | | | | | |
| --- | --- | --- | --- | --- | --- | --- | --- | --- | --- |
|  |  | Cummulative Incidence Rate | | | | Case Fathality Rate | | | |
|  |  | (Symptomatic Cases of COVID-19/Estimated Population)*100 000 | | | | (Confirmed COVID-19 death/Symptomatic Cases of COVID-19)*100 | | | |
|  |  | Jan-April | May | June | As of June 20, 2020 | Jan-April | May 1st to 30 | June 1st to 20, 2020 | As of June 20, 2020 |
|  | Quintile | IRR (95%CI) | IRR (95%CI) | IRR (95%CI) | IRR (95%CI) | IRR (95%CI) | IRR (95%CI) | IRR (95%CI) | IRR (95%CI) |
|  | n | 28304 | 81116 | 65683 | 175148 | 3301 | 10408 | 7064 | 20773 |
| Proportion of population aged 60 year or older.^a^ | 1 | Ref. | Ref. | Ref. | Ref. | Ref. | Ref. | Ref. | Ref. |
|  | 2 | 1.12 (0.92, 1.35). | 1.06 (0.91, 1.22) | **1.16 (1.01, 1.34)** | 1.11 (0.98, 1.26) | 1.27 (0.97, 1.64) | 1.06 (0.89, 1.27) | 1.02 (0.84, 1.22) | 1.07 (0.92, 1.24) |
|  | 3 | 1.21 (0.97, 1.5) | 1.16 (0.98, 1.36) | 1.14 (0.97, 1.34) | **1.18 (1.03, 1.36)** | 1.25 (0.9, 1.73) | 1.13 (0.92, 1.4) | 1 (0.81, 1.25) | 1.15 (0.96, 1.37) |
|  | 4 | **1.33 (1.04, 1.71)** | 0.93 (0.77, 1.12) | 1.08 (0.91, 1.3) | 1.06 (0.91, 1.23) | **1.59 (1.08, 2.33)** | 1.09 (0.85, 1.4) | 0.95 (0.73, 1.23) | 1.11 (0.91, 1.36) |
|  | 5 | **1.69 (1.21, 2.35)** | 0.89 (0.7, 1.13) | 1.08 (0.85, 1.37) | 1.07 (0.88, 1.3) | **2.43 (1.37, 4.29)** | 1.28 (0.88, 1.85) | 1.09 (0.74, 1.61) | 1.25 (0.93, 1.68) |
| Proportion of males.^a^ | 1 | Ref. | Ref. | Ref. | Ref. | Ref. | Ref. | Ref. | Ref. |
|  | 2 | 0.9 (0.72, 1.13) | 1.04 (0.87, 1.24) | 1.06 (0.9, 1.26) | 1 (0.87, 1.16) | 1.04 (0.73, 1.49) | 1.06 (0.85, 1.32) | 1.1 (0.87, 1.41) | 1.01 (0.84, 1.22) |
|  | 3 | 0.8 (0.63, 1.01) | 0.89 (0.74, 1.07) | 1.05 (0.88, 1.26) | 0.91 (0.78, 1.07) | 1.14 (0.78, 1.66) | 1 (0.79, 1.26) | 1.03 (0.79, 1.33) | 0.96 (0.79, 1.17) |
|  | 4 | **0.68 (0.52, 0.87)** | 0.98 (0.81, 1.19) | 1.09 (0.91, 1.32) | 0.94 (0.8, 1.1) | 0.96 (0.64, 1.44) | 1.05 (0.81, 1.35) | 1.1 (0.84, 1.45) | 1 (0.81, 1.23) |
|  | 5 | **0.74 (0.54, 1)** | 1.14 (0.9, 1.43) | 0.91 (0.73, 1.14) | 0.92 (0.76, 1.11) | 1.22 (0.76, 1.97) | 1.2 (0.88, 1.64) | 1.12 (0.8, 1.56) | 1.13 (0.88, 1.46) |
| Diabetes prevalence in adults aged 20 years or older.^b^ | 1 | Ref. | Ref. | Ref. | Ref. | Ref. | Ref. | Ref. | Ref. |
|  | 2 | 1.26 (1, 1.6) | 0.97 (0.8, 1.16) | 1.07 (0.89, 1.29) | 1.05 (0.9, 1.22) | **2.04 (1.44, 2.9)** | 1.21 (0.96, 1.53) | 1.11 (0.88, 1.42) | **1.3 (1.08, 1.58)** |
|  | 3 | 1.2 (0.91, 1.58) | **1.28 (1.04, 1.59)** | **1.29 (1.05, 1.59)** | **1.27 (1.06, 1.52)** | **2.49 (1.62, 3.83)** | **2.13 (1.6, 2.84)** | **1.98 (1.48, 2.66)** | **2.01 (1.6, 2.54)** |
|  | 4 | 1.25 (0.9, 1.74) | **1.46 (1.12, 1.88)** | 1.15 (0.89, 1.48) | 1.22 (0.98, 1.52) | **2.99 (1.81, 4.95)** | **3.09 (2.21, 4.32)** | **2.1 (1.49, 2.98)** | **2.6 (1.97, 3.43)** |
|  | 5 | **1.84 (1.36, 2.5)** | **1.68 (1.33, 2.14)** | 1.19 (0.94, 1.5) | **1.39 (1.13, 1.7)** | **2.93 (1.85, 4.64)** | **2.37 (1.75, 3.2)** | **1.59 (1.16, 2.19)** | **2.14 (1.66, 2.75)** |
| Obesity prevalence in adults aged 20 years or older.^b^ | 1 | Ref. | Ref. | Ref. | Ref. | Ref. | Ref. | Ref. | Ref. |
|  | 2 | 1.08 (0.85, 1.38) | **0.72 (0.6, 0.88)** | **0.71 (0.59, 0.86)** | **0.77 (0.66, 0.91)** | 1.08 (0.74, 1.58) | **0.7 (0.54, 0.9)** | **0.55 (0.43, 0.72)** | **0.71 (0.58, 0.88)** |
|  | 3 | 1.19 (0.89, 1.58) | **0.72 (0.58, 0.9)** | 0.85 (0.69, 1.04) | 0.85 (0.71, 1.02) | 1.45 (0.95, 2.24) | **0.74 (0.56, 0.98)** | 0.94 (0.7, 1.24) | 0.9 (0.71, 1.13) |
|  | 4 | 0.91 (0.66, 1.27) | **0.62 (0.48, 0.81)** | 0.81 (0.63, 1.03) | **0.72 (0.58, 0.9)** | 0.66 (0.4, 1.07) | **0.5 (0.36, 0.7)** | **0.66 (0.47, 0.94)** | **0.61 (0.46, 0.8)** |
|  | 5 | 4 (2.67, 6.01) | **1.94 (1.4, 2.71)** | **2.33 (1.71, 3.18)** | **2.37 (1.8, 3.12)** | **3.56 (1.96, 6.45)** | **1.52 (1.01, 2.27)** | **1.82 (1.19, 2.78)** | **1.93 (1.37, 2.71)** |
| Hypertension prevalence in adults aged 20 years or older.^b^ | 1 | Ref. | Ref. | Ref. | Ref. | Ref. | Ref. | Ref. | Ref. |
|  | 2 | 0.94 (0.69, 1.29) | 0.86 (0.67, 1.11) | 0.94 (0.74, 1.2) | 0.9 (0.73, 1.11) | **0.49 (0.31, 0.79)** | **0.52 (0.38, 0.73)** | **0.6 (0.43, 0.84)** | **0.59 (0.45, 0.78)** |
|  | 3 | **0.59 (0.45, 0.78)** | **0.75 (0.61, 0.93)** | 0.93 (0.75, 1.14) | 0.84 (0.7, 1.01) | **0.34 (0.22, 0.53)** | **0.58 (0.44, 0.78)** | **0.62 (0.46, 0.82)** | **0.58 (0.46, 0.74)** |
|  | 4 | 0.79 (0.58, 1.08) | 1.06 (0.83, 1.36) | 1.17 (0.92, 1.48) | 1.14 (0.92, 1.39) | **0.32 (0.2, 0.52)** | **0.65 (0.47, 0.9)** | **0.51 (0.37, 0.71)** | **0.55 (0.42, 0.71)** |
|  | 5 | **0.53 (0.38, 0.73)** | **0.61 (0.47, 0.78)** | **0.63 (0.49, 0.82)** | **0.62 (0.5, 0.76)** | **0.28 (0.17, 0.45)** | **0.42 (0.3, 0.59)** | **0.42 (0.29, 0.59)** | **0.4 (0.31, 0.53)** |
| Crude mortality rate of diabetes in adults 20 years or older.^c^ | 1 | Ref. | Ref. | Ref. | Ref. | Ref. | Ref. | Ref. | Ref. |
|  | 2 | 0.81 (0.64, 1.04) | 1 (0.83, 1.2) | **1.2 (1.01, 1.44)** | 1.05 (0.91, 1.22) | 1.01 (0.67, 1.51) | 1.08 (0.82, 1.4) | **1.34 (1.01, 1.79)** | 1.17 (0.94, 1.45) |
|  | 3 | 0.98 (0.77, 1.26) | 0.98 (0.81, 1.18) | **1.22 (1.01, 1.46)** | 1.05 (0.91, 1.23) | 1.22 (0.81, 1.85) | **1.35 (1.03, 1.76)** | **1.58 (1.19, 2.11)** | **1.34 (1.07, 1.66)** |
|  | 4 | 0.91 (0.7, 1.18) | 1.06 (0.87, 1.28) | **1.4 (1.16, 1.69)** | 1.16 (1, 1.36) | 1.54 (0.99, 2.38) | **1.56 (1.18, 2.06)** | **1.78 (1.32, 2.4)** | **1.54 (1.23, 1.94)** |
|  | 5 | 1.12 (0.85, 1.48) | 1.14 (0.93, 1.4) | **1.4 (1.14, 1.7)** | **1.23 (1.04, 1.45)** | **1.75 (1.09, 2.8)** | **1.56 (1.16, 2.11)** | **1.69 (1.22, 2.34)** | **1.58 (1.24, 2.01)** |
| Crude mortality rate of hypertension in adults 20 years or older.^c^ | 1 |  |  |  |  |  |  |  |  |
|  | 3 | 0.97 (0.8, 1.19) | 0.92 (0.78, 1.07) | 1.04 (0.9, 1.21) | 1 (0.87, 1.14) | 0.96 (0.7, 1.33) | 0.99 (0.81, 1.21) | 0.85 (0.69, 1.05) | 0.94 (0.8, 1.11) |
|  | 4 | 0.96 (0.79, 1.16) | 1.05 (0.91, 1.21) | 1.01 (0.88, 1.17) | 1.02 (0.91, 1.15) | 0.87 (0.62, 1.21) | 1.08 (0.89, 1.32) | 0.92 (0.75, 1.14) | 1.02 (0.87, 1.2) |
|  | 5 | 0.96 (0.77, 1.19) | 0.99 (0.85, 1.16) | **0.86 (0.74, 1)** | 0.93 (0.83, 1.06) | 1 (0.66, 1.52) | 0.91 (0.7, 1.16) | 1.05 (0.81, 1.36) | 1.03 (0.85, 1.25) |
| Crude mortality rate of cerebrovascular disease in adults 20 years or older.^c^ | 1 |  |  |  |  |  |  |  |  |
|  | 2 | 0.84 (0.64, 1.1) | 0.87 (0.71, 1.07) | 0.98 (0.8, 1.2) | 0.91 (0.76, 1.08) | 1.02 (0.63, 1.64) | 0.89 (0.67, 1.18) | 0.96 (0.7, 1.3) | 0.95 (0.75, 1.19) |
|  | 3 | **0.76 (0.6, 0.97)** | 0.97 (0.82, 1.16) | 1.07 (0.9, 1.27) | 0.98 (0.85, 1.13) | 1.06 (0.68, 1.65) | 0.94 (0.72, 1.21) | 1.1 (0.83, 1.46) | 1.01 (0.82, 1.24) |
|  | 4 | **0.77 (0.61, 0.97)** | 0.91 (0.77, 1.07) | 1.09 (0.93, 1.28) | 0.96 (0.83, 1.09) | 0.92 (0.59, 1.43) | 0.91 (0.7, 1.18) | 1.32 (1, 1.74) | 1.04 (0.85, 1.27) |
|  | 5 | **0.73 (0.56, 0.93)** | 0.94 (0.79, 1.11) | 0.97 (0.81, 1.15) | 0.91 (0.79, 1.05) | 0.8 (0.47, 1.34) | 0.94 (0.7, 1.25) | 0.95 (0.68, 1.32) | 0.93 (0.74, 1.17) |
| Proportion of the population in extreme poverty | 1 |  |  |  |  |  |  |  |  |
|  | 2 | 1.13 (0.9, 1.42) | **1.24 (1.04, 1.48)** | 0.93 (0.78, 1.11) | 1.07 (0.92, 1.25) | 1.38 (0.98, 1.95) | 1.08 (0.85, 1.36) | 0.88 (0.7, 1.12) | 1.04 (0.86, 1.27) |
|  | 3 | 1.3 (0.95, 1.77) | **1.28 (1, 1.62)** | 0.94 (0.74, 1.19) | 1.11 (0.9, 1.35) | **1.67 (1.01, 2.78)** | 0.96 (0.69, 1.33) | 1.07 (0.77, 1.5) | 1.1 (0.84, 1.44) |
|  | 4 | 0.87 (0.58, 1.29) | 1.17 (0.86, 1.58) | 1.03 (0.77, 1.37) | 1.08 (0.85, 1.39) | 1.75 (0.86, 3.57) | 0.92 (0.6, 1.41) | 1.41 (0.9, 2.19) | 1.21 (0.86, 1.7) |
|  | 5 | **0.56 (0.32, 0.97)** | 0.92 (0.63, 1.36) | 0.71 (0.48, 1.03) | 0.81 (0.59, 1.11) | 1.19 (0.41, 3.49) | 0.87 (0.48, 1.58) | 0.91 (0.47, 1.75) | 1 (0.63, 1.6) |
| Proportion of illiterate population aged 5 year or older.^e^ | 1 |  |  |  |  |  |  |  |  |
|  | 2 | **0.65 (0.53, 0.81)** | **0.73 (0.61, 0.87)** | **0.71 (0.6, 0.84)** | **0.72 (0.62, 0.83)** | 0.76 (0.55, 1.04) | **0.78 (0.62, 0.96)** | **0.78 (0.62, 0.97)** | **0.75 (0.63, 0.9)** |
|  | 3 | **0.54 (0.41, 0.73)** | **0.64 (0.51, 0.8)** | **0.64 (0.51, 0.8)** | **0.63 (0.53, 0.77)** | **0.62 (0.39, 0.98)** | **0.73 (0.54, 0.98)** | **0.68 (0.5, 0.93)** | **0.68 (0.53, 0.87)** |
|  | 4 | **0.49 (0.34, 0.69)** | **0.61 (0.47, 0.79)** | **0.46 (0.36, 0.6)** | **0.55 (0.44, 0.68)** | 0.61 (0.34, 1.11) | **0.64 (0.44, 0.92)** | **0.56 (0.38, 0.83)** | **0.62 (0.46, 0.84)** |
|  | 5 | 0.8 (0.5, 1.28) | **0.5 (0.36, 0.7)** | **0.38 (0.28, 0.53)** | **0.5 (0.38, 0.65)** | 0.93 (0.39, 2.24) | 0.68 (0.41, 1.13) | **0.47 (0.27, 0.81)** | **0.62 (0.42, 0.92)** |
| Proportion of indigenous population aged 5 year or older.^e^ | 1 |  |  |  |  |  |  |  |  |
|  | 2 | 1.09 (0.89, 1.34) | 0.94 (0.81, 1.11) | **0.84 (0.72, 0.98)** | 0.91 (0.8, 1.04) | 1.35 (0.96, 1.91) | **1.75 (1.39, 2.21)** | **1.31 (1.04, 1.65)** | **1.43 (1.2, 1.71)** |
|  | 3 | **1.31 (1.05, 1.63)** | 1.01 (0.85, 1.21) | **0.84 (0.72, 1)** | 0.94 (0.81, 1.08) | **1.95 (1.36, 2.8)** | **2.27 (1.77, 2.91)** | **1.45 (1.14, 1.86)** | **1.68 (1.38, 2.03)** |
|  | 4 | 0.85 (0.66, 1.1) | 0.83 (0.69, 1) | **0.75 (0.63, 0.9)** | **0.77 (0.66, 0.9)** | 1.25 (0.81, 1.92) | **1.91 (1.45, 2.51)** | 1.22 (0.93, 1.62) | **1.39 (1.13, 1.73)** |
|  | 5 | 0.97 (0.68, 1.4) | 0.79 (0.61, 1.03) | **0.65 (0.51, 0.84)** | **0.7 (0.57, 0.86)** | 0.99 (0.5, 1.97) | **1.75 (1.18, 2.6)** | 1.01 (0.66, 1.55) | 1.18 (0.87, 1.61) |
| Proportion of population economically active aged 12 year or older.^d^ | 1 |  |  |  |  |  |  |  |  |
|  | 2 | 1.28 (0.93, 1.78) | **1.26 (1.02, 1.55)** | 1.09 (0.88, 1.34) | **1.18 (1, 1.4)** | 1.57 (0.76, 3.24) | 1.38 (0.93, 2.04) | 0.99 (0.66, 1.49) | 1.22 (0.92, 1.63) |
|  | 3 | 1.34 (0.95, 1.9) | **1.49 (1.19, 1.88)** | 1.15 (0.92, 1.45) | **1.38 (1.15, 1.66)** | 1.9 (0.9, 4.02) | **1.51 (1, 2.27)** | 0.9 (0.59, 1.39) | 1.29 (0.95, 1.74) |
|  | 4 | 1.29 (0.88, 1.88) | **1.48 (1.15, 1.91)** | 1.21 (0.94, 1.55) | **1.42 (1.16, 1.74)** | 1.88 (0.86, 4.12) | 1.46 (0.95, 2.25) | 1 (0.63, 1.57) | 1.35 (0.97, 1.86) |
|  | 5 | 1.45 (0.95, 2.2) | **1.77 (1.33, 2.36)** | 1.27 (0.96, 1.69) | **1.61 (1.28, 2.04)** | 2.27 (0.99, 5.23) | 1.51 (0.95, 2.42) | 1.12 (0.68, 1.83) | **1.5 (1.06, 2.14)** |
| Rate of economic units that operate essential activities during COVID-19 outbreakd | 1 |  |  |  |  |  |  |  |  |
|  | 2 | 1.2 (0.94, 1.54) | **1.49 (1.25, 1.77)** | **1.34 (1.12, 1.59)** | **1.41 (1.22, 1.62)** | 1.19 (0.76, 1.88) | 1.51 (1.16, 1.97) | 1.23 (0.92, 1.65) | **1.33 (1.08, 1.64)** |
|  | 3 | 1.09 (0.84, 1.41) | **1.42 (1.17, 1.71)** | **1.26 (1.05, 1.51)** | **1.34 (1.15, 1.56)** | 0.94 (0.59, 1.5) | 1.23 (0.93, 1.62) | 1.18 (0.87, 1.59) | 1.21 (0.97, 1.5) |
|  | 4 | 1.2 (0.92, 1.57) | **1.62 (1.34, 1.97)** | **1.64 (1.35, 1.99)** | **1.6 (1.36, 1.88)** | 1.07 (0.67, 1.73) | **1.62 (1.22, 2.14)** | **1.71 (1.26, 2.32)** | **1.54 (1.23, 1.93)** |
|  | 5 | 1.12 (0.83, 1.51) | **1.41 (1.14, 1.75)** | **1.53 (1.24, 1.89)** | **1.48 (1.24, 1.77)** | 0.75 (0.44, 1.28) | **1.29 (0.94, 1.76)** | **1.42 (1.02, 1.98)** | **1.29 (1.01, 1.66)** |
| Population density^c^ | 1 |  |  |  |  |  |  |  |  |
|  | 2 | 1.26 (0.93, 1.71) | 1.23 (0.99, 1.53) | 1.18 (0.96, 1.45) | **1.2 (1.01, 1.43)** | 1.13 (0.65, 1.95) | **1.53 (1.07, 2.18)** | **1.84 (1.28, 2.64)** | **1.58 (1.21, 2.07)** |
|  | 3 | 1.16 (0.85, 1.58) | **1.39 (1.1, 1.74)** | 1.14 (0.92, 1.41) | **1.23 (1.02, 1.47)** | 1.42 (0.83, 2.43) | **1.84 (1.29, 2.62)** | **1.66 (1.15, 2.4)** | **1.67 (1.27, 2.19)** |
|  | 4 | **1.56 (1.12, 2.17)** | **1.97 (1.54, 2.51)** | **1.57 (1.25, 1.96)** | **1.7 (1.4, 2.07)** | **1.76 (1.01, 3.05)** | **2.39 (1.65, 3.46)** | **2.38 (1.63, 3.47)** | **2.28 (1.71, 3.04)** |
|  | 5 | **1.8 (1.24, 2.6)** | **2.39 (1.81, 3.15)** | **1.95 (1.51, 2.51)** | **2.08 (1.67, 2.6)** | **1.87 (1.04, 3.38)** | **3.1 (2.09, 4.6)** | **2.64 (1.75, 3.97)** | **2.72 (1.99, 3.73)** |
| Proportion of houses with availability of clear water inside the domicile.^d^ | 1 |  |  |  |  |  |  |  |  |
|  | 2 | **0.78 (0.64, 0.94)** | **0.76 (0.65, 0.89)** | 0.88 (0.76, 1.03) | **0.81 (0.71, 0.93)** | 0.87 (0.66, 1.16) | 0.86 (0.71, 1.05) | 0.82 (0.67, 1.01) | 0.85 (0.72, 1) |
|  | 3 | 0.87 (0.7, 1.08) | 0.85 (0.72, 1.01) | 1.07 (0.91, 1.27) | 0.93 (0.81, 1.07) | 1.1 (0.81, 1.5) | 1.04 (0.83, 1.3) | 1.05 (0.84, 1.32) | 1.01 (0.84, 1.21) |
|  | 4 | 0.99 (0.78, 1.25) | 1.07 (0.89, 1.29) | 1.16 (0.97, 1.39) | 1.1 (0.94, 1.29) | 0.98 (0.7, 1.39) | 1.08 (0.85, 1.37) | 1.12 (0.87, 1.43) | 1.04 (0.86, 1.27) |
|  | 5 | 1.03 (0.79, 1.36) | 1.12 (0.91, 1.37) | **1.46 (1.19, 1.79)** | **1.27 (1.07, 1.51)** | 1.15 (0.74, 1.78) | **1.39 (1.05, 1.82)** | 1.18 (0.88, 1.58) | 1.24 (0.99, 1.55) |
| Proportion of houses without sewage drainage.^d^ | 1 |  |  |  |  |  |  |  |  |
|  | 2 | 1.16 (0.95, 1.42) | 0.97 (0.82, 1.14) | 0.99 (0.85, 1.15) | 1.01 (0.88, 1.15) | 0.86 (0.64, 1.15) | 0.92 (0.75, 1.12) | 0.89 (0.73, 1.1) | 0.93 (0.78, 1.09) |
|  | 3 | 0.93 (0.73, 1.19) | 0.98 (0.81, 1.18) | 1.01 (0.84, 1.21) | 1.03 (0.88, 1.2) | 0.74 (0.5, 1.08) | 0.84 (0.66, 1.08) | 1.02 (0.79, 1.33) | 0.94 (0.77, 1.16) |
|  | 4 | 1.04 (0.78, 1.38) | 0.96 (0.78, 1.19) | 0.89 (0.72, 1.1) | 0.95 (0.8, 1.14) | 0.69 (0.43, 1.12) | 0.91 (0.68, 1.21) | 1.01 (0.75, 1.37) | 0.96 (0.76, 1.22) |
|  | 5 | 0.86 (0.59, 1.26) | 0.84 (0.64, 1.09) | **0.74 (0.57, 0.96)** | **0.8 (0.65, 1)** | 0.71 (0.35, 1.42) | 0.75 (0.51, 1.1) | 0.68 (0.44, 1.05) | 0.75 (0.55, 1.02) |
| Proportion of houses with dirt floor.^d^ | 1 |  |  |  |  |  |  |  |  |
|  | 2 | 1.09 (0.89, 1.34) | 1.13 (0.96, 1.33) | 1.11 (0.95, 1.3) | 1.12 (0.98, 1.29) | 0.98 (0.72, 1.33) | **1.25 (1.01, 1.54)** | 1.13 (0.92, 1.4) | **1.21 (1.01, 1.44)** |
|  | 3 | 1.05 (0.81, 1.37) | 1.24 (1.02, 1.52) | **1.3 (1.07, 1.59)** | 1.25 (1.05, 1.48) | 1.09 (0.73, 1.62) | 1.23 (0.93, 1.61) | 1.08 (0.82, 1.43) | 1.21 (0.97, 1.52) |
|  | 4 | 0.76 (0.55, 1.06) | 0.98 (0.77, 1.24) | 1.17 (0.92, 1.49) | 1.03 (0.84, 1.26) | 0.66 (0.39, 1.11) | 1 (0.72, 1.4) | 0.96 (0.67, 1.35) | 0.97 (0.74, 1.27) |
|  | 5 | 0.66 (0.43, 1.01) | 1 (0.74, 1.35) | **1.45 (1.09, 1.95)** | 1.09 (0.85, 1.39) | 0.49 (0.22, 1.11) | 0.88 (0.57, 1.37) | 0.8 (0.5, 1.28) | 0.8 (0.56, 1.13) |
| Rate of Hospitals/population | 1 |  |  |  |  |  |  |  |  |
|  | 4 | **1.24 (1.03, 1.5)** | **1.19 (1.03, 1.38)** | **1.23 (1.07, 1.41)** | **1.25 (1.1, 1.41)** | 1.26 (0.93, 1.71) | 1.17 (0.97, 1.41) | **1.3 (1.07, 1.58)** | **1.25 (1.07, 1.45)** |
|  | 5 | **1.25 (1.03, 1.5)** | 1.14 (0.99, 1.31) | 1.13 (0.99, 1.3) | **1.16 (1.03, 1.3)** | 1.25 (0.91, 1.72) | 0.99 (0.81, 1.2) | 1.09 (0.89, 1.34) | 1.06 (0.9, 1.24) |
| Rate of units of Primary Health Care Services/population | 1 |  |  |  |  |  |  |  |  |
|  | 2 | 0.93 (0.77, 1.11) | 0.95 (0.82, 1.11) | 0.98 (0.84, 1.13) | 0.97 (0.85, 1.1) | 1.17 (0.89, 1.53) | 1.06 (0.89, 1.27) | 0.97 (0.81, 1.17) | 1.04 (0.9, 1.21) |
|  | 3 | **0.74 (0.59, 0.94)** | 0.91 (0.77, 1.09) | 0.93 (0.78, 1.11) | 0.9 (0.77, 1.04) | 0.99 (0.68, 1.44) | 0.93 (0.74, 1.17) | 1.02 (0.8, 1.31) | 0.96 (0.79, 1.16) |
|  | 4 | 0.83 (0.62, 1.11) | 1.06 (0.86, 1.31) | **1.26 (1.02, 1.54)** | 1.15 (0.97, 1.37) | 1.03 (0.62, 1.73) | 1.05 (0.77, 1.43) | 1.08 (0.77, 1.5) | 1.07 (0.84, 1.37) |
|  | 5 | 0.79 (0.53, 1.19) | 1.05 (0.8, 1.39) | 1.12 (0.85, 1.47) | 1.12 (0.89, 1.4) | 0.71 (0.31, 1.67) | 0.91 (0.56, 1.48) | 1.17 (0.71, 1.95) | 1.03 (0.72, 1.49) |
| Rate of Social Assistance medical units/population | 1 |  |  |  |  |  |  |  |  |
|  | 4 | **1.3 (1.04, 1.62)** | 1.11 (0.92, 1.34) | 1.06 (0.89, 1.27) | 1.08 (0.91, 1.27) | 1.14 (0.85, 1.54) | 1.19 (0.97, 1.45) | 0.95 (0.77, 1.17) | 1.06 (0.89, 1.26) |
|  | 5 | **1.25 (1.05, 1.48)** | **1.47 (1.28, 1.68)** | **1.36 (1.2, 1.55)** | **1.35 (1.2, 1.51)** | 1.17 (0.9, 1.53) | **1.29 (1.08, 1.53)** | 1.07 (0.9, 1.28) | 1.14 (0.99, 1.31) |
| Proportion of without health care insurance^d^ | 1 |  |  |  |  |  |  |  |  |
|  | 2 | 0.87 (0.69, 1.09) | **0.7 (0.58, 0.84)** | **0.7 (0.59, 0.84)** | **0.71 (0.61, 0.83)** | 0.81 (0.57, 1.17) | **0.78 (0.62, 0.97)** | **0.64 (0.5, 0.81)** | **0.71 (0.59, 0.86)** |
|  | 3 | 0.77 (0.57, 1.04) | **0.57 (0.46, 0.73)** | **0.65 (0.52, 0.82)** | **0.62 (0.51, 0.75)** | 0.99 (0.61, 1.6) | **0.58 (0.43, 0.78)** | **0.55 (0.4, 0.76)** | **0.6 (0.47, 0.77)** |
|  | 4 | 1.02 (0.72, 1.45) | **0.57 (0.44, 0.74)** | **0.67 (0.51, 0.86)** | **0.63 (0.51, 0.79)** | 1.21 (0.66, 2.24) | 0.77 (0.54, 1.11) | **0.55 (0.37, 0.82)** | **0.7 (0.52, 0.94)** |
|  | 5 | 1.13 (0.74, 1.71) | **0.64 (0.47, 0.86)** | **0.7 (0.52, 0.94)** | **0.7 (0.55, 0.9)** | **2.54 (1.2, 5.38)** | **0.59 (0.37, 0.94)** | **0.47 (0.29, 0.78)** | **0.64 (0.45, 0.92)** |
| Proportion of population affiliated to the Mexican Social Security Institute^d^ | 1 |  |  |  |  |  |  |  |  |
|  | 2 | 0.91 (0.66, 1.25) | 0.94 (0.76, 1.16) | 0.99 (0.8, 1.22) | 0.95 (0.8, 1.13) | 0.67 (0.35, 1.28) | 0.94 (0.66, 1.34) | 0.84 (0.56, 1.25) | 0.86 (0.66, 1.13) |
|  | 3 | 1.22 (0.87, 1.73) | 0.85 (0.67, 1.07) | 0.99 (0.78, 1.25) | 0.95 (0.79, 1.15) | 1.29 (0.65, 2.57) | 1.08 (0.73, 1.58) | 0.89 (0.58, 1.37) | 1.02 (0.76, 1.38) |
|  | 4 | 1.08 (0.74, 1.57) | 0.81 (0.62, 1.06) | 0.88 (0.68, 1.15) | 0.87 (0.7, 1.07) | 1.32 (0.64, 2.75) | 0.95 (0.62, 1.44) | 0.88 (0.55, 1.4) | 0.97 (0.7, 1.35) |
|  | 5 | 1.18 (0.77, 1.83) | **0.72 (0.53, 0.99)** | 0.79 (0.58, 1.08) | 0.8 (0.62, 1.04) | 1.34 (0.59, 3.01) | 0.81 (0.5, 1.3) | 0.74 (0.44, 1.23) | 0.83 (0.57, 1.21) |
| Proportion of population with Private Health Insurance^d^ | 1 |  |  |  |  |  |  |  |  |
|  | 2 | **1.57 (1.12, 2.19)** | 1.2 (0.96, 1.49) | 1.11 (0.9, 1.37) | **1.2 (1.01, 1.42)** | 0.97 (0.47, 1.96) | 1.05 (0.74, 1.5) | 1.08 (0.73, 1.62) | 1.05 (0.8, 1.38) |
|  | 3 | **1.43 (1.01, 2.02)** | **1.46 (1.16, 1.83)** | **1.3 (1.05, 1.61)** | **1.37 (1.15, 1.63)** | 1.35 (0.67, 2.74) | 1.05 (0.73, 1.52) | 1.31 (0.87, 1.96) | 1.16 (0.88, 1.53) |
|  | 4 | 1.21 (0.85, 1.73) | 1.15 (0.91, 1.46) | 1.23 (0.98, 1.54) | 1.17 (0.97, 1.41) | 1.27 (0.61, 2.62) | 0.86 (0.59, 1.25) | 1.09 (0.72, 1.65) | 0.98 (0.73, 1.31) |
|  | 5 | 1.34 (0.92, 1.95) | **1.34 (1.04, 1.72)** | 1.24 (0.97, 1.57) | **1.28 (1.05, 1.56)** | 1.47 (0.7, 3.08) | 0.91 (0.61, 1.34) | 1.06 (0.69, 1.63) | 1.01 (0.75, 1.37) |

Bold numbers indicates statistically significant in comparison with quintile 1 (p<0.05). Cummulative Incidence rate was defined as the confirmed symptomatic cases in the municipality’s estimated population. Case-fathality rate was defined as the confirmed deaths in confirmed symptomatic cases. ^a^Municipality data of 2020, ^b^State data of 2018, ^c^Municipality data of 2018, ^d^Municipality data of 2015, ^e^Municipality data of 2010, ^f^Municipality data of 2019. ^£^Open data from the Ministry of Health. The sample includes 175 148 confirmed cases and 20773 deaths as of June 20th, 2020. Database was downloaded the 21th of June, 2020.
